# Cure of syngeneic carcinomas with targeted IL-12 through obligate reprogramming of lymphoid and myeloid immunity

**DOI:** 10.1172/jci.insight.157448

**Published:** 2022-03-08

**Authors:** Youji Hong, Yvette Robbins, Xinping Yang, Wojciech K. Mydlarz, Anastasia Sowers, James B. Mitchell, James L. Gulley, Jeffrey Schlom, Sofia R. Gameiro, Cem Sievers, Clint T. Allen

**Affiliations:** 1 Section on Translational Tumor Immunology, National Institute on Deafness and Other Communication Disorders, NIH, Bethesda, Maryland, USA.; 2 Department of Otolaryngology-Head and Neck Surgery, Johns Hopkins School of Medicine, Baltimore, Maryland, USA.; 3 Radiation Biology Branch,; 4 Geniturinary Malignancy Branch, Center for Cancer Research, and; 5 Laboratory of Tumor Immunology and Biology, National Cancer Institute, NIH, Bethesda, Maryland, USA.

**Keywords:** Immunology, Oncology, Adaptive immunity, Cytokines, Immunotherapy

## Abstract

Therapeutic IL-12 has demonstrated the ability to reduce local immune suppression in preclinical models, but clinical development has been limited by severe inflammation-related adverse events with systemic administration. Here, we show that potent immunologic tumor control of established syngeneic carcinomas can be achieved by i.t. administration of a tumor-targeted IL-12 antibody fusion protein (NHS–rmIL-12) using sufficiently low doses to avoid systemic toxicity. Single-cell transcriptomic analysis and ex vivo functional assays of NHS–rmIL-12–treated tumors revealed reinvigoration and enhanced proliferation of exhausted CD8^+^ T lymphocytes, induction of Th1 immunity, and a decrease in Treg number and suppressive capacity. Similarly, myeloid cells transitioned toward inflammatory phenotypes and displayed reduced suppressive capacity. Cell type–specific IL-12 receptor–KO BM chimera studies revealed that therapeutic modulation of both lymphoid and myeloid cells is required for maximum treatment effect and tumor cure. Study of single-cell data sets from human head and neck carcinomas revealed IL-12 receptor expression patterns similar to those observed in murine tumors. These results describing the diverse mechanisms underlying tumor-directed IL-12–induced antitumor immunity provide the preclinical rationale for the clinical study of i.t. NHS–IL-12.

## Introduction

The immunosuppressive tumor microenvironment (TME) established by cells of myeloid and lymphoid origin can promote tumorigenesis by protecting cancer cells from effective antitumor immunity. Tregs suppress effector cell function by producing antiinflammatory cytokines, such as TGF-β or IL-10, or by CTLA-4–mediated competition for activating cell surface ligands on professional antigen presenting cells (1). Similarly, the presence of immunosuppressive myeloid cells within the TME, such as M2 tumor–associated macrophages or myeloid-derived suppressor cells (MDSCs), has been linked to enhanced disease aggressiveness (2). Therapeutic interventions that interfere with these various immunosuppressive mechanisms may enhance antitumor immunity.

IL-12 is a proinflammatory cytokine that exerts pleiotropic effects on different cells of the immune system. For instance, IL-12 polarizes CD4^+^ T cells toward an IFN-γ–producing Th1 phenotype and counteracts Treg function (3, 4). IL-12 has consistently demonstrated considerable antitumor activity in preclinical carcinoma models (5–7). However, the clinical development of IL-12 as a systemic therapeutic for patients with advanced cancer has been hampered by the observation of severe adverse effects in early clinical studies (8, 9). To address this limitation, alternate IL-12 delivery strategies aimed at limiting systemic exposure have been developed. One such approach, NHS–IL-12 consists of 2 therapeutic IL-12 molecules conjugated to the histone-specific antibody NHS76. This fusion protein targets IL-12 to regions of extracellular DNA fragments generally present within necrotic sites of tumors (10). Early clinical study of NHS–IL-12 administered s.c. in patients with relapsed solid cancers demonstrated a manageable toxicity profile up to a maximally tolerated dose and immune-related clinical activity sufficient to warrant continued development (11).

The development of antitumor immunity with s.c. NHS–IL-12 treatment is dose dependent, with increased doses of drug leading to greater tumor control (12). Insufficient IL-12 concentrations within the TME achieved with maximally tolerated s.c. dosing of NHS–IL-12 may be limiting the development of antitumor immunity sufficient to control tumors in patients (13). We hypothesized that further targeting the TME with i.t. injection of NHS–IL-12 would lead to increased local concentrations and enhance antitumor immunity while decreasing systemic exposure. Here, we demonstrate greater tumor control with i.t. NHS–recombinant murine IL-12 (rmIL-12) compared with peripheral s.c. administration in multiple syngeneic carcinoma models. In an immunogenic oral cancer model that demonstrated robust tumor control or cure with i.t. NHS–rmIL-12 treatment, we utilized single-cell transcriptomic analysis to dissect the mechanisms underlying reversal of local immune suppression and induction of systemic antitumor immunity. Within the Treg compartment, we observed a reduction in cell frequency and suppressive capacity, which was accompanied by an increase in the number of Th1 cells and evidence of reinvigoration across a range of exhausted CD8^+^ T lymphocyte subsets. Similarly, within the myeloid compartment of the TME, we observed a transition from predominantly immunosuppressive cell types, such as M2- and monocytic-MDSC-like cells, toward proinflammatory M1-like cells. Notably, BM chimera studies demonstrated that direct IL-12 modulation of T cells alone was insufficient to achieve maximum treatment effect leading to cure of tumors. These findings support that targeting IL-12 to the TME with NHS–IL-12 and i.t. injection enhances antitumor immunity through multiple concomitant mechanisms and provides the scientific rationale for the clinical study of i.t. NHS–IL-12.

## Results

### Intratumoral NHS–rmIL-12 exhibits high antitumor efficacy.

**Figure 1:**
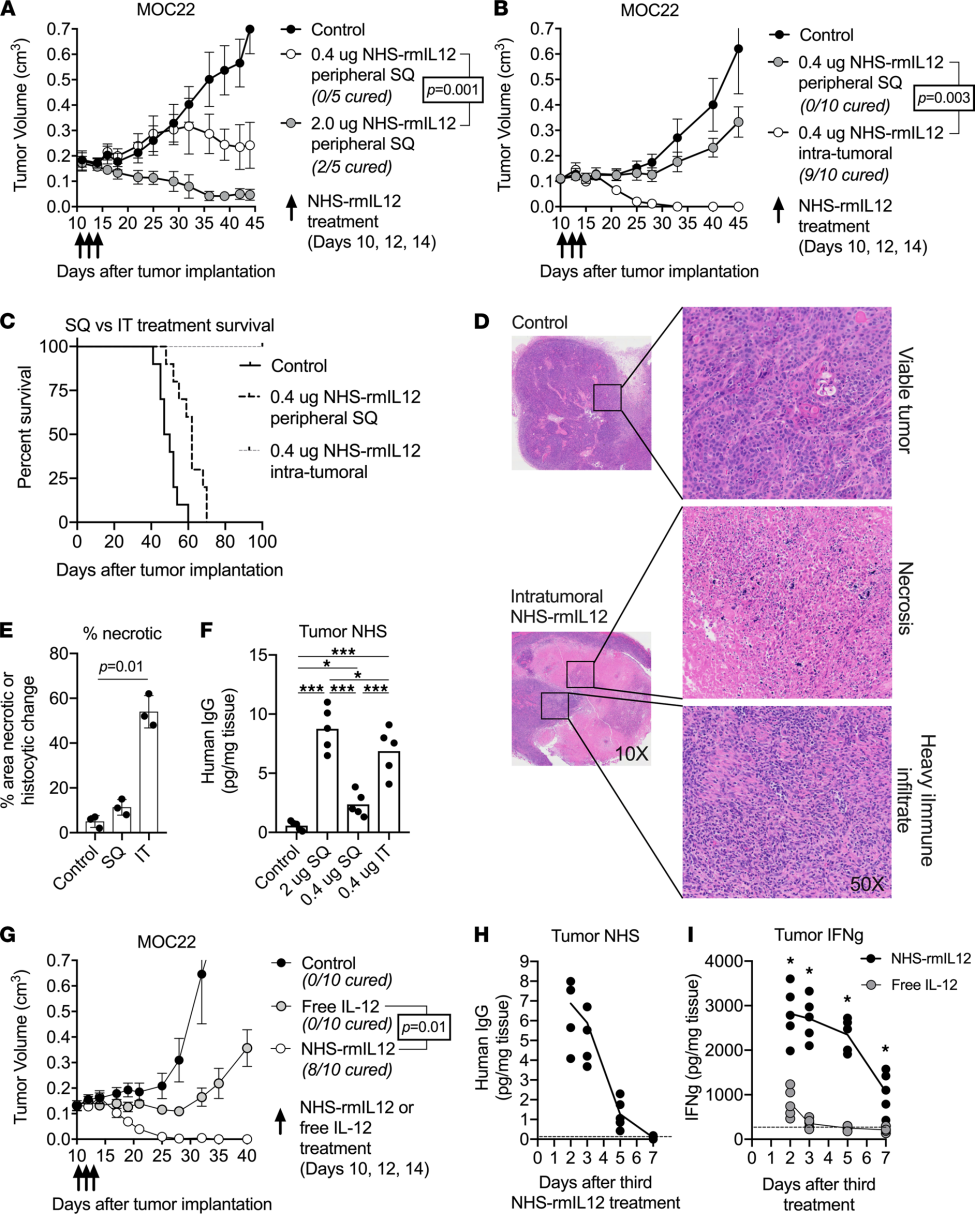
Dose-reduced tumor targeted NHS–rmIL-12 eradicates established oral cancers. (**A**) WT B6 mice (*n* = 5/group) bearing established MOC22 carcinomas were treated with PBS control, high-dose (2.0 μg) or low-dose (0.4 μg) peripheral s.c. NHS–rmIL-12. Significance determined by 2-way ANOVA. (**B**) Mice (*n* = 10/group) bearing established MOC22 tumors were treated with 3 doses of PBS control or peripheral s.c. or i.t. low-dose NHS–rmIL-12. Significance determined by 2-way ANOVA. (**C**) Survival curve of MOC22 tumor–bearing mice (*n* = 15/group) treated with peripheral s.c. or i.t. low-dose NHS–rmIL-12 over 3 independent experiments. (**D**) Forty-eight hours after the third PBS control or low-dose i.t. NHS–rmIL-12 treatment, tumors (*n* = 3/group) were harvested, stained with H&E, and assessed for histologic changes via microscopy. Focal areas of interest from 10× magnification photomicrographs enlarged to 50× magnification are shown. (**E**) The percentage of tumor area necrosis in PBS control or low-dose peripheral s.c. or i.t. NHS–rmIL-12–treated tumors (*n* = 3/group) was quantified via digital annotation in QuPath. Significance determined by 1-way ANOVA. (**F**) Forty-eight hours after the third PBS control, peripheral s.c. high- or low-dose or i.t. low-dose NHS–rmIL-12 treatment, MOC22 tumors (*n* = 5/group) were harvested and digested, and human IgG concentrations were measured from tumor supernatant via ELISA. Significance determined by 1-way ANOVA. (**G**) Mice (*n* = 10/group) bearing established MOC22 tumors were treated with PBS control, i.t. low-dose NHS–rmIL-12, or dose equivalent (0.29 μg) free IL-12. Significance determined by 2-way ANOVA. (**H**) A time course of human IgG concentration was measured in MOC22 tumor supernatants (*n* = 5/group) following 3 low-dose i.t. NHS–rmIL-12 treatments. The dashed horizontal line represents human IgG levels in MOC22 tumors treated with PBS control. (**I**) A time course of IFN-γ concentration was measured in MOC22 tumor supernatants (*n* = 5/group) via ELISA following 3 low-dose i.t. NHS–rmIL-12or free IL-12 treatments. Asterisks indicate a significant difference (*P* < 0.05) between NHS–rmIL-12 and free IL-12 determined by a 2-tailed Student *t* test. The dashed horizontal line represents human IgG levels in MOC22 tumors treated with PBS control. *P < 0.05; ***P < 0.001.

To better characterize treatment effects of NHS–rmIL-12, we employed a mouse tumor model induced by s.c. injection of mouse oral cancer 22 (MOC22) carcinogen–induced syngeneic oral cavity carcinoma cells. Treatment of resulting mice bearing MOC22 carcinomas with 3 doses of peripheral s.c. 2.0 μg NHS–rmIL-12 (high dose) resulted in significant growth delay or cure. To further limit systemic exposure, we also evaluated treatment with a reduced dose of 0.4 μg (low dose). Although low-dose NHS–rmIL-12 displayed substantially reduced treatment toxicity compared with high-dose NHS–rmIL-12, as indicated by the lack of body weight reduction during treatment observed with high-dose NHS–rmIL-12 (Supplemental Figure 1; supplemental material available online with this article; https://doi.org/10.1172/jci.insight.157448DS1), we also observed a concomitant, significant reduction in treatment efficacy in the low-dose treatment regime (Figure 1A). Next, we evaluated if treatment efficacy of low-dose NHS–rmIL-12 could be improved by direct tumor targeting using i.t. injection. Low-dose i.t. NHS–rmIL-12 resulted in 90% cure rates (Figure 1B) and significantly improved overall survival (Figure 1C). Similarly improved treatment efficacy with i.t. NHS–rmIL-12, compared with s.c. NHS–rmIL-12, was observed in 2 additional syngeneic models of oral (MOC1) and colon (MC38) carcinoma (Supplemental Figure 2, A and B).

Histologic assessment of tumors 2 days after treatment with low-dose i.t. NHS–rmIL-12 revealed a significant degree of tumor necrosis and immune infiltrate compared with tumors treated with PBS control or low-dose s.c. NHS–rmIL-12 (Figure 1, D and E). Consistent with the enhanced treatment response, we observed increased NHS concentrations in tumors treated with low-dose i.t. NHS–rmIL-12, at levels comparable with those achieved with high-dose s.c. NHS–rmIL-12 (Figure 1F). To assess the importance of NHS-mediated targeting of rmIL-12 to the TME, we compared NHS–rmIL-12 to free cytokine by treating mice bearing MOC22 tumors with either low-dose NHS–rmIL-12 or molar-equivalent free rmIL-12. Free rmIL-12 treatment induced modest tumor growth delay followed by disease progression compared with 80% cure observed with NHS–rmIL-12 (Figure 1G). A time course analysis of tumor NHS concentration following treatment with low-dose i.t. NHS–rmIL-12 revealed persistence of drug in the TME for 3–5 days (Figure 1H). Comparison of tumor IFN-γ concentrations revealed a significantly elevated and more durable IFN-γ increase following i.t. NHS–rmIL-12 compared with treatment with i.t. free rmIL-12 (Figure 1I). Conversely, serum IFN-γ concentrations were greater in mice treated with i.t. free rmIL-12 compared with treatment with NHS–rmIL-12, potentially indicating an increased risk of systemic toxicity (Supplemental Figure 3A). Serum concentrations of NHS–rmIL-12 following systemic administration were undetectable as early as 48 hours after treatment (Supplemental Figure 3B), indicating that low levels of the drug reach systemic circulation following i.t. treatment. Cumulatively, these data suggest that low-dose i.t. NHS–rmIL-12 (hereafter, low-dose i.t. NHS-rmIL-12 is referred to as NHS-rmIL-12) results in greater antitumor control compared with low-dose s.c. NHS–rmIL-12 or i.t. free rmIL-12, likely through retention of drug in the TME resulting in greater production of IFN-γ.

### Single-cell RNA-Seq reveals expression changes consistent with an ongoing treatment response and elevated IFN-γ levels.

**Figure 2:**
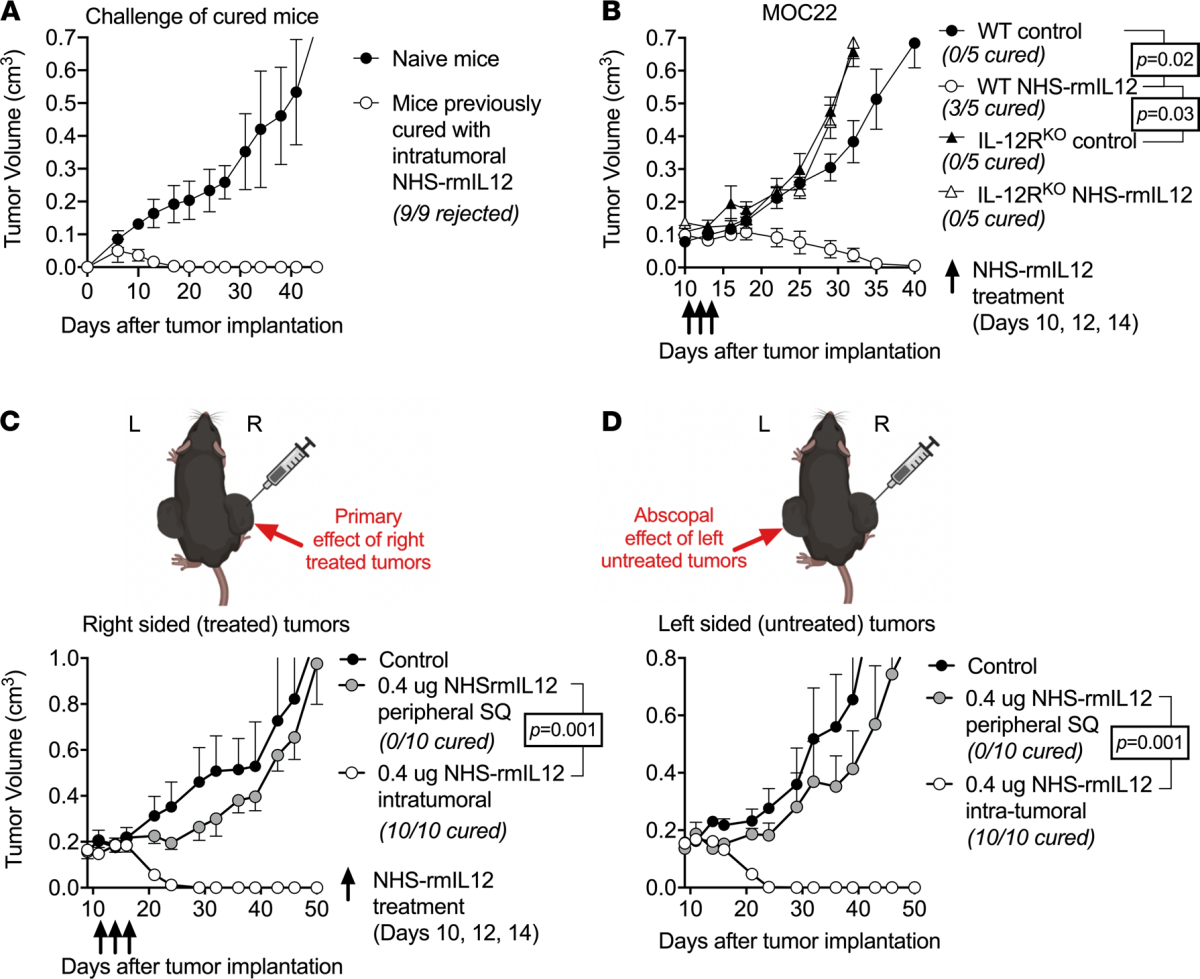
NHS–rmIL-12 treatment leads to abscopal control of distant tumors through IL-12R-dependent immunity. (**A**) Naive WT B6 mice or mice that rejected MOC22 tumors after i.t. NHS–rmIL-12 treatment were challenged with MOC22 and followed for tumor growth. (**B**) WT B6 mice or IL-12Rb2–deficient (IL-12R^KO^) mice bearing established MOC22 tumors (*n* = 5/group) were treated with PBS control or low-dose i.t. NHS–rmIL-12. Significance determined by 2-way ANOVA. (**C** and **D**) Mice bearing established MOC22 tumors in bilateral flanks were treated with unilateral (right sided) low-dose i.t. NHS–rmIL-12, and both the treated (**C**) tumor and the untreated (**D**) tumors were followed for growth. Significance determined by 2-way ANOVA.

To explore the role of the immune system in the NHS–rmIL-12 treatment response, we evaluated the formation of immunologic memory in mice that were cured following NHS–rmIL-12 treatment. Whereas MOC22 tumors readily engrafted in naive mice, tumors failed to form in mice cured following treatment, indicating the presence of immunological memory (Figure 2A). To rule out the possibility that NHS–rmIL-12 directly alters the viability of MOC22 tumor cells in vivo, NHS–rmIL-12 treatment was performed in *IL-12Rb2*–KO mice bearing established MOC22 tumors. Genetic deletion of the IL-12 receptor (IL-12R) on host cells accelerated the growth of MOC22 tumors in untreated mice and totally abrogated the treatment response to NHS–rmIL-12 (Figure 2B). To verify the lack of direct NHS–rmIL-12–mediated cytotoxicity, MOC22 cells in culture were exposed to drug at concentrations similar to in vivo levels and assayed for survival and proliferation via real-time impedance analysis. NHS–rmIL-12 treatment did not alter MOC22 cell viability (Supplemental Figure 4). Furthermore, exposure to NHS–rmIL-12 neither enhanced the killing of MOC22 cells by tumor infiltrating lymphocytes (TIL) cultured from MOC22 tumors nor altered cytotoxicity of these cultured TIL. These results suggest that NHS–rmIL-12 does not directly affect MOC22 tumor cells and enhances antitumor immunity through its effect on host cells.

Considering the development of immunological memory, we further characterized systemic immunity in response to NHS–rmIL-12 treatment. Unilateral low-dose i.t. NHS–rmIL-12 treatment of mice bearing bilateral flank MOC22 tumors resulted in 100% cure of both treated (Figure 2C) and untreated (Figure 2D) tumors. Comparatively, treatment with low-dose s.c. NHS–rmIL-12 resulted in modest growth delay only. These results established that i.t. NHS–rmIL-12 treatment results in host cell IL-12R–dependent induction of systemic antitumor immunity sufficient to control locally treated, as well a distant untreated, tumors.

### Single-cell RNA-Seq reveals expression changes consistent with an IFN-γ response.

**Figure 3:**
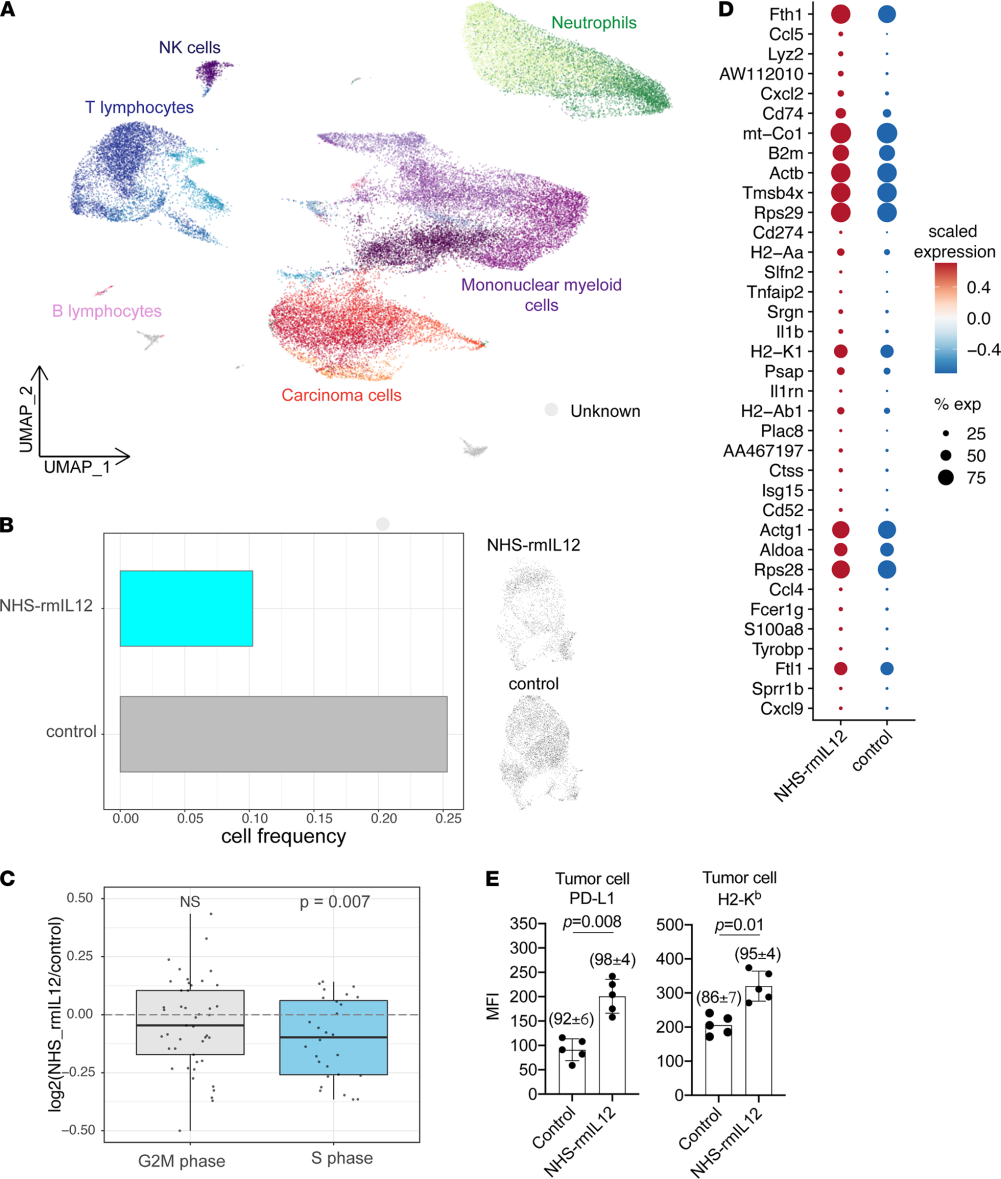
NHS–rmIL-12 treatment results in reduced viability and enhanced immunogenicity of tumor cells. (**A**) UMAP embedding shows 44,036 single cells obtained 48 hours after the third PBS control or NHS–rmIL-12 treatment from 2 tumors per condition. Cell types were identified using clustering and marker gene expression analysis (Supplemental Figure 5). (**B**) Bar graph shows fraction of carcinoma cells in tumors treated with PBS control or NHS–rmIL-12 (left panel). UMAP embedding shows carcinoma cells from tumors treated with PBS control or NHS–rmIL-12 (right panel). (**C**) Box plot show log_2_-transformed fold changes in average expression of G2M- or S phase–associated genes comparing NHS–rmIL-12– and control-treated carcinoma cells. Wilcoxon signed-rank test was used to determine statistical significance. (**D**) Dot plot shows expression of genes upregulated (adjusted *P* ≤ 0.05) in NHS–rmIL-12-–treated carcinoma cells. Circle color corresponds to scaled average expression; circle size denotes fraction of cells with nonzero gene expression of corresponding gene. (**E**) Bar graph shows median fluorescence intensity (MFI) of cell surface PD-L1 and MHC class I (H2-K^b^) on CD45^–^CD31^–^PDGFR^–^ tumor cells measured by flow cytometry 48 hours after treatment with NHS–rmIL-12 or PBS control. *P* value determined by Student’s *t* test.

To explore the mechanisms underlying the development of systemic antitumor immunity following NHS–rmIL-12 treatment, single-cell transcriptomic analysis of control and treated tumors was performed. Clustering of single cells combined with marker gene expression analysis revealed distinct carcinoma, lymphoid, and myeloid cell subsets (Figure 3A and Supplemental Figure 5). Comparison of carcinoma cell abundance between different treatment conditions revealed a decrease in the fraction of carcinoma cells following NHS–rmIL-12 treatment, consistent with an early treatment response (Figure 3B). Furthermore, we observed a reduced expression of S phase–associated genes following treatment, suggesting that remaining cells are less proliferative (Figure 3C). Assessment of genes most upregulated within NHS–rmIL-12–treated carcinoma revealed increased expression of IFN-γ–responsive genes involved in antigen presentation, including *B2m*, *H2-A*, *Cd74*, the immune checkpoint *Cd274*, and the T lymphocyte chemokine *Cxcl9* (Figure 3D). Increased protein level expression of cell-surface PD-L1 and H2-K^b^ was validated using flow cytometry of dissociated control and treated tumors (Figure 3E). These single-cell analyses of carcinoma cells within MOC22 tumors supports early loss of tumor cell viability and enhanced tumor cell immunogenicity through increased expression of IFN-γ–responsive genes following treatment with i.t. NHS–rmIL-12.

### NHS–rmIL-12 treatment leads to lymphocyte polarization and enhanced cytotoxicity.

**Figure 4:**
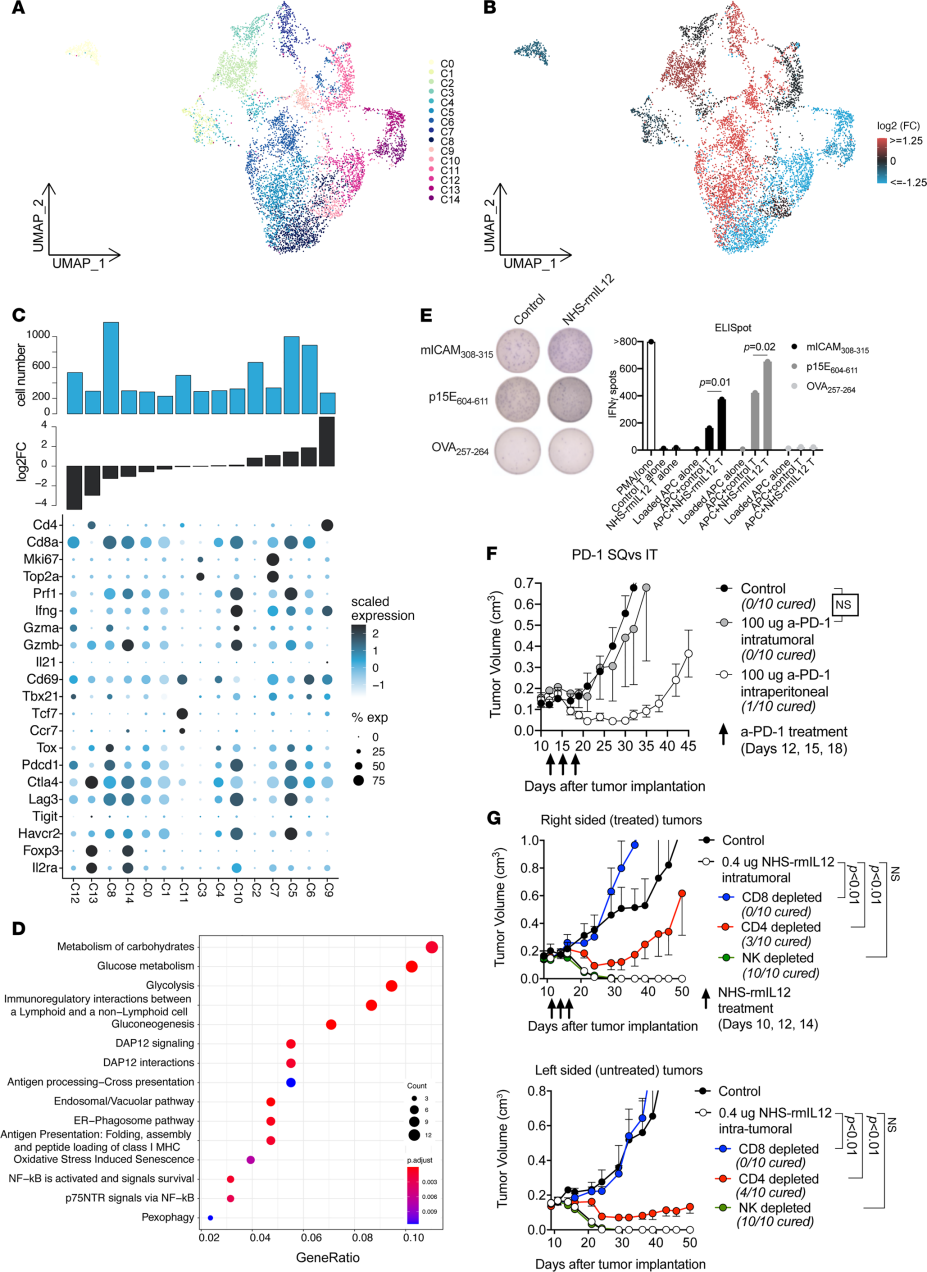
NHS–rmIL-12 promotes Th1 immunity and reinvigorates exhausted antigen-specific CD8^+^ T lymphocytes. (**A**) UMAP embedding of T lymphocytes colored by cluster identity. (**B**) UMAP embedding of T lymphocytes; clusters were colored by log_2_ fold change in relative cell abundance in NHS–rmIL-12– and PBS control-treated tumors. Red represents a relative increase, and blue a relative decrease in cell frequency following treatment with NHS–rmIL-12. (**C**) Dot plot showing expression of select T lymphocyte–related genes across T lymphocyte clusters sorted by fold change in relative cell abundance comparing cells from NHS–rmIL-12– to control-treated tumors (lower bar graph). Circle color corresponds to scaled average expression; circle size denotes fraction of cells with nonzero gene expression of corresponding gene. Top bar graph represents total cell number. (**D**) Dot plot showing Reactome terms enriched in genes upregulated by NHS–rmIL-12 treatment within cells of clusters 5 and 8. (**E**) Antigen specificity of cultured TIL from tumors treated with NHS–rmIL-12 or PBS control following coculture with antigen-presenting cells pulsed with the neoepitope mICAM_308-315_, shared epitope p15E_604-611_, or control epitope OVA_257-264_, measured via ELISpot assay. Significance determined by 1-way ANOVA. (**F**) Mice (*n* = 10/group) bearing established MOC22 tumors were treated with 3 doses of i.t. or i.p. PD-1 mAb clone RPM1-14 (100 μg) or isotype (rat IgG2a) control and followed for tumor growth. Significance determined by 2-way ANOVA. (**G**) Mice bearing established MOC22 tumors in bilateral flanks were treated with unilateral (right sided) low-dose i.t. NHS–rmIL-12, in the presence or absence of CD8-, CD4-, or NK1.1-depleted antibodies (200 μg i.p. twice weekly for 3 weeks each), and both the treated (right sided) tumor and the untreated (left sided) tumors were followed for growth. Significance determined by 2-way ANOVA.

To explore functional and phenotypic changes within T lymphocytes following treatment, corresponding cells were further subclustered into 15 distinct cell clusters (Figure 4A). We observed substantial variability within the T lymphocyte compartment; multiple cell subsets displayed profound increase or depletion during NHS–rmIL-12 treatment, respectively (Figure 4B). To better understand treatment-related differences, we sorted individual cell clusters by fold increase during treatment and evaluated marker gene expression at the cluster level (Figure 4C). Two prominent CD4^+^ clusters displayed opposite fold changes following NHS–rmIL-12 treatment. A substantial reduction in *Foxp3*, *Il2r*a, and *Ctla4* expressing Treg-like cells (cluster 13) accompanied by an increase in Th1-like cells positive for *Ifng* and *Il21* (cluster 9) was observed, suggesting polarization of the CD4 compartment away from immunosuppressive and toward Th1 helper function.

The 10 distinct CD8^+^ subsets could be broadly classified as *Mki67*^+^ and *Top2a*^+^ proliferating (T_PROL_, clusters 3 and 7), exhaustion marker positive exhausted (T_EX_), or *Tcf7*^+^ stem-like (T_STEM_, cluster 11) subsets. Consistent with the gene expression profile of tumor antigen-specific CD8^+^ T lymphocytes in human tumors (14), the majority of all CD8^+^ subsets displayed an exhausted profile with expression of *Tox*, *Pdcd1*, *CTLA4*, *Lag3*, and *Havcr2*. However, greater expression of effector molecules *Prf1*, *Gzma*, *Gzmb*, and *Ifng* was observed in T_EX_ subsets that were gained following NHS–rmIL-12 treatment (clusters 5 and 10) compared with T_EX_ subsets that were reduced (clusters 0, 1, 8, and 12). A T_PROL_ subset (cluster 7) was also enriched following NHS–rmIL-12 treatment. A T_STEM_ subset (cluster 11) was not significant altered, suggesting that activation of stem-like cells may not play a role at this early time point after treatment. Differential gene expression analysis between 2 T_EX_ subsets (clusters 5 and 8) that represented the 2 most frequent clusters among all T lymphocyte clusters revealed enrichment of Reactome pathway terms related to glucose metabolism, DAP12 signaling, and antigen presentation following treatment with NHS–rmIL-12 (Figure 4D). Increased accumulation of PD-1^+^CD8^+^ T lymphocytes expressing CD107a as a measure of antigen engagement and degranulation following treatment with NHS–rmIL-12 was validated using flow cytometry of fresh dissociated tumors (Supplemental Figure 6).

Coculture of cultured TIL from control or NHS–rmIL-12–treated tumors with irradiated splenocytes pulsed with MOC22 tumor antigen was performed to explore their antigen specificity (Figure 4E). Consistent with the increased expression of effector molecules (Figure 4C), greater numbers of CD8^+^ T cells specific for the previously identified MHC class I–restricted MOC22-specific neoantigen mICAM_308-315_ and the shared antigen p15E_604-611_ were identified in TIL from treated tumors compared with control, indicating that reinvigorated T_EX_ subsets enriched after NHS–rmIL-12 treatment may be tumor antigen specific.

The MOC22 model is sensitive to therapeutic PD-1 immune checkpoint blockade when administered early after tumor engraftment (15). Given evidence of PD-L1 expression within the TME of MOC22 tumors and PD-1 expression on TIL, we tested whether tumor control could also be achieved with i.t. PD-1 mAb treatment of established MOC22 tumors. Although systemic administration of PD-1 mAb resulted in significant tumor delay, treatment with i.t. PD-1 mAb demonstrated no effect (Figure 4F). These results indicate that i.t. NHS–rmIL-12 treatment resulted in greater treatment efficacy compared with i.t. PD pathway immune checkpoint blockade.

NK cell clustering revealed distinct subclusters that were low in overall frequency and displayed differential abundances following NHS–rmIL-12 treatment (Supplemental Figure 7, A and B). NK cells from treated tumors displayed greater expression of activation markers *Prf1*, *Gzma*, and *IFNg* and were enriched for Reactome pathway terms related to DAP12 signaling, previously demonstrated to be critical for NK cell effector function (ref. 16 and Supplemental Figure 7, C–E). To further assess the importance of CD8^+^ and CD4^+^ T lymphocyte as well as NK cells in vivo, we performed respective cell depletion experiments and evaluated response to NHS–rmIL-12. Although depletion of CD8^+^ cells totally abrogated NHS–rmIL-12 treatment response in treated and distant tumors, depletion of CD4^+^ cells had a less pronounced effect with intermediate cure rates (Figure 4G). Depletion of NK cells did not alter treatment efficacy. Together, these results indicate that NHS–rmIL-12 results in robust CD8-dependent antitumor immunity, and that CD4^+^ cells are necessary for maximum treatment effect, possibly through Th1 function, but insufficient to control tumors alone.

### NHS–rmIL-12 treatment reduces suppressive capacity of Tregs.

**Figure 5:**
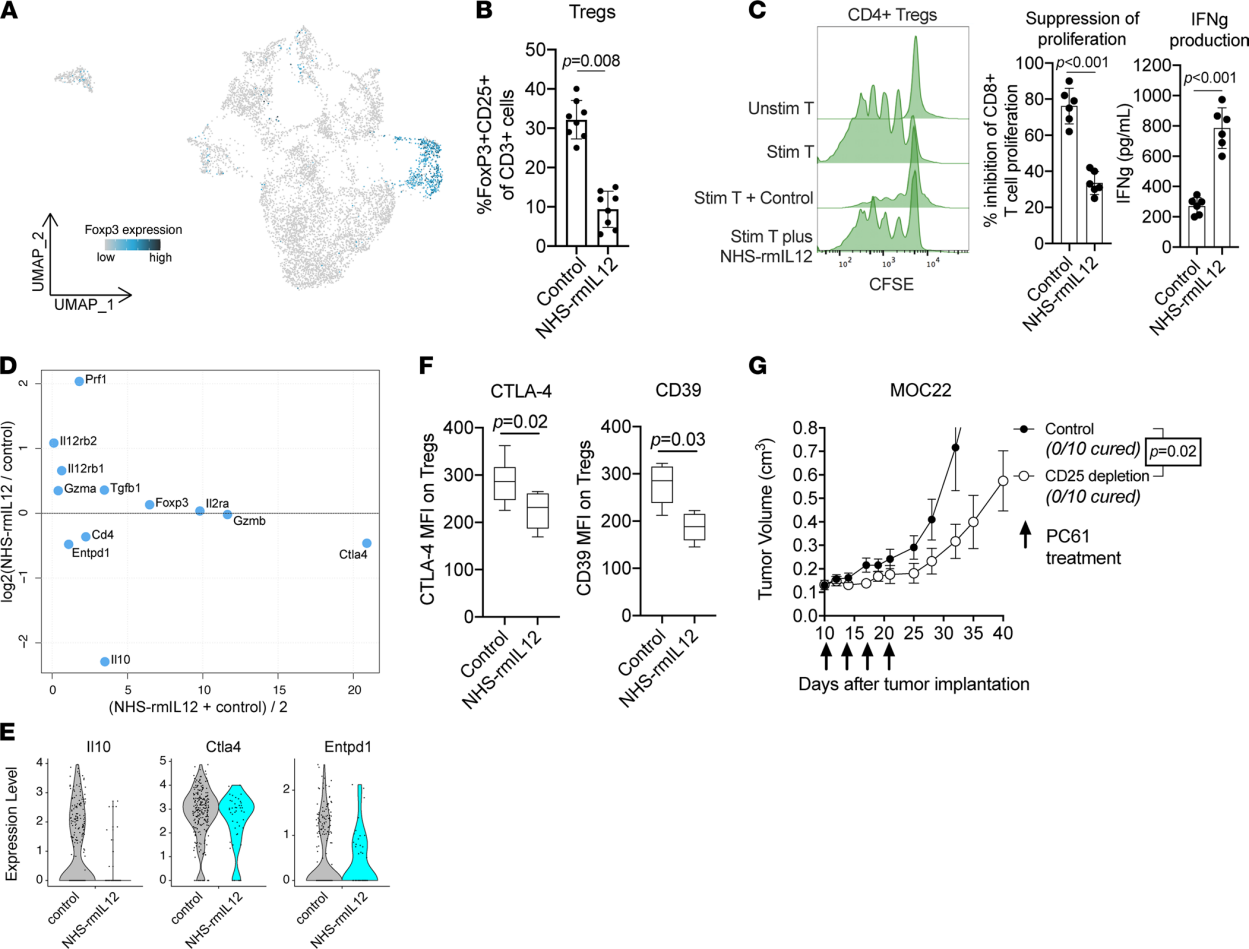
NHS–rmIL-12 decreases the frequency and suppressive capacity of Tregs. (**A**) UMAP embedding of T lymphocytes colored by expression of *FoxP3*. (**B**) Quantification of FoxP3^+^ and CD25^+^ CD3^+^ T lymphocytes from tumors treated with NHS–rmIL-12 or PBS control measured by flow cytometry. *P* value determined by Student’s *t* test. (**C**) CD4^+^ and CD25^+^ cells were isolated from tumors treated with NHS–rmIL-12 or PBS control via magnetic selection and cocultured with CFSE-labeled WT T lymphocytes stimulated with CD3/CD28 antibodies. Representative CFSE histograms are shown, T lymphocyte proliferation was measured by flow cytometry, and IFN-γ production was measured by ELISA. *P* value determined by Student’s *t* test. (**D**) Dot plot showing fold change in average expression of Treg-related genes, comparing Tregs from NHS–rmIL-12– and control-treated tumors, over mean gene expression. (**E**) Violin plots of *CTLA4*, *Entpd1,* and *IL10* expression in CD4^+^, FoxP3^+^, and CD25^+^ cells from tumors treated with NHS–rmIL-12 or PBS control. (**F**) Forty-eight hours after treatment with NHS–rmIL-12 or PBS control, MFI of cell surface CTLA-4 or CD39 on FoxP3^+^ and CD25^+^ CD4^+^ T lymphocytes was measured by flow cytometry. *P* value determined by Student’s *t* test. (**G**) Mice (*n* = 10/group) bearing established MOC22 tumors were treated with 4 doses of the anti-CD25 mAb clone PC61 (200 μg) or isotype control (rat IgG1) and followed for tumor growth. Significance determined by 1-way ANOVA.

We further validated the reduction in *FoxP3^+^* and *Il2ra*^+^ Tregs, observed in our single-cell analysis of NHS–rmIL-12–treated tumors (Figure 5A), using flow cytometry (Figure 5B). In addition, we assessed the suppressive capacity of Tregs isolated from NHS–rmIL-12– and control-treated tumors. Interestingly, we observed a marked reduction in the ability of freshly isolated CD4^+^ and CD25^+^ Tregs from NHS–rmIL-12–treated tumors to suppress autologous T lymphocyte proliferation and IFN-γ production compared with Tregs isolated from control tumors (Figure 5C). To better characterize the mechanism underlying the differences in suppressive capacity, we compared the expression of genes known to play a role in Treg function between NHS–rmIL-12– and control-treated Tregs (Figure 5D). This comparison revealed increased expression of Prf1 and the 2 IL-12R subunits (Il12rb1 and Il12Rb2) in NHS–rmIL-12–treated Tregs, which are transcriptional target genes downstream of IL-12 CITE. While Treg lineage markers Foxp3 and Il2ra showed similar expression, we observed reduced expression of *Il10*, *Ctla4*, and *Entpd1* (Figure 5, D and E). Reduced expression of cell surface CTLA-4 and CD39 (encoded by *Entpd1*) on Tregs was validated using flow cytometry (Figure 5F). Given these results, we explored whether Treg depletion alone in mice bearing established MOC22 tumors using a CD25 antibody was sufficient to induce tumor control. CD25 depletion only modestly delayed MOC22 tumor growth (Figure 5G). In summary, these data suggest that NHS–rmIL-12 reduces both the number and suppressive capacity of Tregs, but that abrogation of Tregs alone is insufficient to induce complete disease control observed with NHS–rmIL-12 treatment.

### NHS–rmIL-12 treatment induces polarization of myeloid cells toward inflammatory phenotypes.

**Figure 6:**
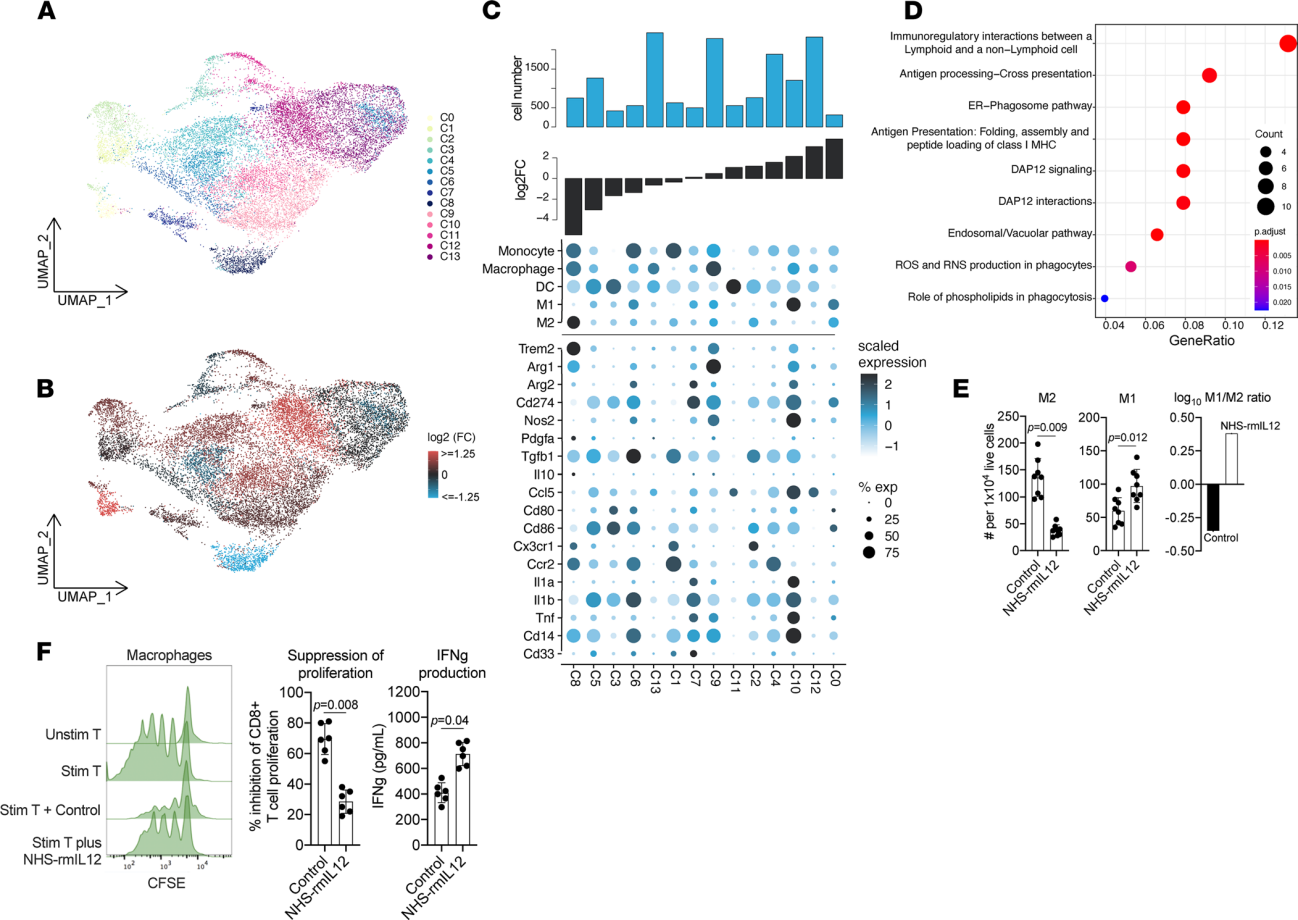
NHS–rmIL-12 reduces immunosuppressive capacity of macrophages. (**A**) UMAP embedding of mononuclear myeloid cells colored by cluster identity. (**B**) UMAP embedding of mononuclear myeloid cells; clusters were colored by log_2_ fold change in relative cell abundance in NHS–rmIL-12– and PBS control–treated tumors. Red represents a relative increase, and blue a relative decrease in cell frequency following treatment with NHS–rmIL-12. (**C**) Dot plot shows module scores related to myeloid cell subsets (top) and myeloid-related genes (bottom) across myeloid cell clusters sorted by fold change in relative cell abundance comparing cells from NHS–rmIL-12– to control-treated tumors (lower bar graph). Circle color corresponds to scaled average expression; circle size denotes fraction of cells with nonzero gene expression of corresponding gene. Top bar graph represents total cell number. (**D**) Dot plot showing Reactome terms enriched in genes upregulated in cells from NHS–rmIL-12–treated tumors within cells of clusters 9 and 13 compared with control-treated cells. (**E**) Quantification of CD206 high (M2) and low (M1) CD11b, F4/80^+^ (CD11c^–^) myeloid cells from tumors 48 hours after treatment, with NHS–rmIL-12 or PBS control measured by flow cytometry. The log_10_-transformed ratio of M1/M2 cells is shown on the right. *P* value determined by Student’s *t* test. (**F**) CD11b^+^ and F4/80^+^ cells were isolated from tumors treated with NHS–rmIL-12 or PBS control via magnetic selection and cocultured with CFSE-labeled WT T lymphocytes stimulated with CD3/CD28 antibodies. Representative CFSE histograms are shown, T lymphocyte proliferation was measured by flow cytometry, and IFN-γ production was measured by ELISA. *P* value determined by Student’s *t* test.

Additional clustering of mononuclear myeloid cells revealed 14 distinct myeloid subsets (Figure 6A), multiple of which underwent substantial changes in abundance following NHS–rmIL-12 treatment (Figure 6, B and C). Cluster 8 demonstrated the greatest reduction following treatment compared with control and scored high for macrophage and M2 module scores (Supplemental Table 1), as well as expression of *Trem2*, previously associated with immunosuppressive activity and increased tumor growth (17). Similarly, we observed the reduction of monocytic-MDSC-like cells (clusters 6 and 1) upon NHS–rmIL-12 treatment, which express relatively high levels of a monocyte signature and *Tgfb1*. Conversely, cluster 10 increased after treatment and scored high for an M1 module score and displayed increased expression of inflammatory genes *Il1a*, *Il1b*, and *Tnf* and the costimulatory gene *Cd86*. Differential gene expression analysis, contrasting treatment conditions, was performed with 2 clusters (clusters 9, 13) that scored high for macrophage but not clearly for M1 or M2 module scores and were also the most abundant myeloid clusters. In agreement with increased antitumor immunity, we observed enrichment of Reactome pathway terms related to immune cell interactions and antigen presentation following treatment with NHS–rmIL-12 (Figure 6D). Consistent with the observed changes in myeloid subsets, flow cytometry confirmed that M2-macrophages expressing high cell-surface CD206 were decreased while M1-like macrophages expressing low CD206 were increased, significantly shifting TME macrophages toward a predominantly M1 phenotype following NHS–rmIL-12 treatment (Figure 6E). To explore whether these phenotypic changes correlated with a change in suppressive function, macrophages from control or treated tumors were sorted from tumors and assayed for T cell suppressive capacity. Total macrophage populations isolated from NHS–rmIL-12–treated tumors demonstrated reduced ability to suppress proliferation of and IFN-γ production by autologous T lymphocytes compared with macrophages isolated from control tumors (Figure 6F).

Neutrophilic cells were clustered into 3 distinct subclusters, which displayed different abundances following NHS–rmIL-12 treatment (Supplemental Figure 8, A and B). Overall, a modest reduction in neutrophilic cells was observed by flow cytometry (Supplemental Figure 8C). Differential gene expression analysis revealed a decrease in *Cxcr2^+^* and *Arg2*^+^ neutrophilic cells but an increase in *Olr1*^+^ neutrophilic cells expressing high levels of *Tgfb*, previously identified as neutrophilic-myeloid–derived suppressor cells in humans (ref. 18 and Supplemental Figure 8D). Upon study of suppressive function, the cumulative ability of these neutrophilic populations to suppress T lymphocyte proliferation and IFN-γ production was not reduced following i.t. NHS–rmIL-12 treatment (Supplemental Figure 8E). Together, these data suggest that NHS–rmIL-12 treatment results in a reduction of immunosuppressive myeloid cells, such as M2 macrophages and monocytic-MDSCs accompanied by an increase in proinflammatory M1-like cells.

### Tumor cure requires NHS–rmIL-12-mediated modulation of the lymphoid and myeloid compartments.

**Figure 7:**
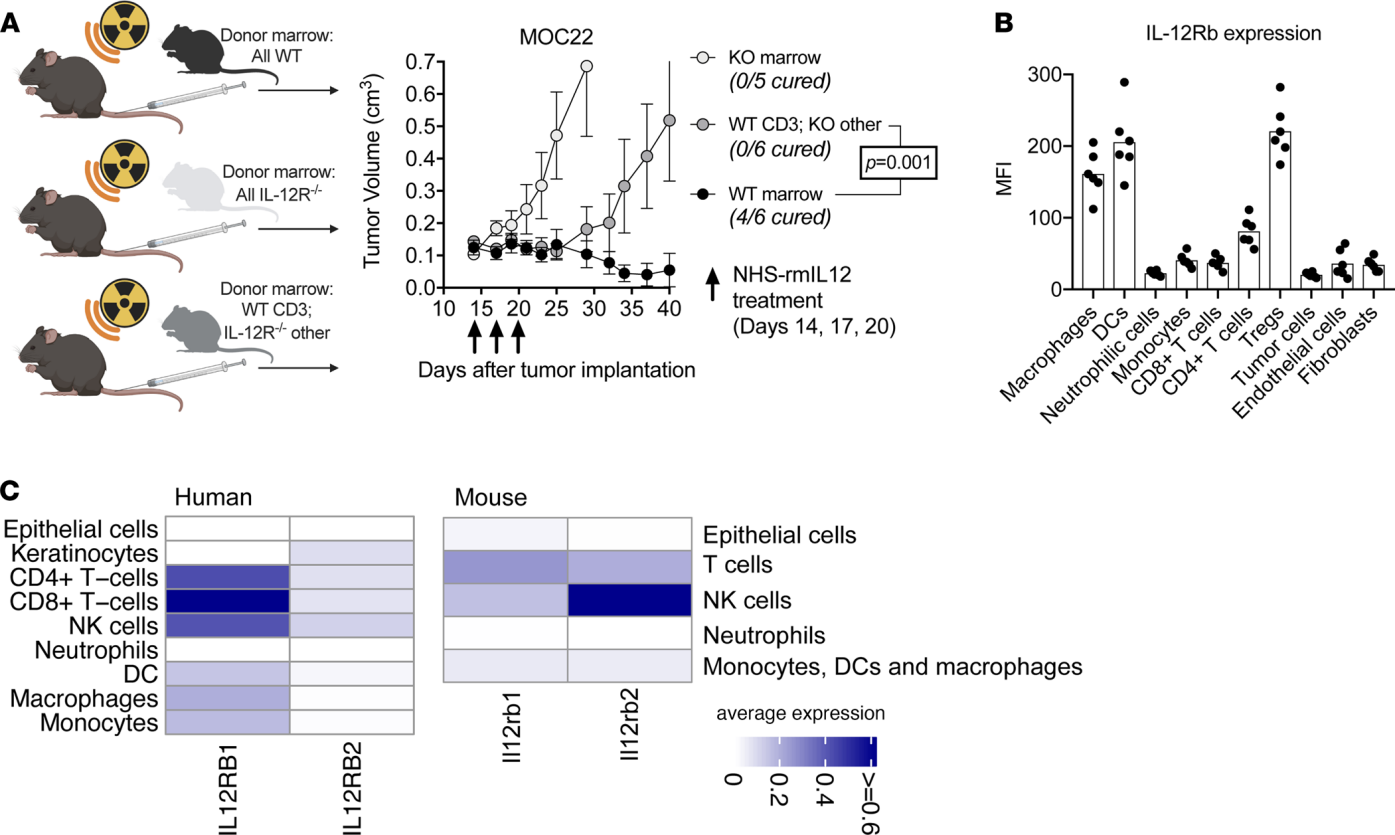
Direct effects of NHS–rmIL-12 on the lymphoid and myeloid compartment are necessary for tumor cure. (**A**) Schematic of chimera experiments (left panel). WT B6 mice were irradiated to 9 Gy and transplanted with WT marrow, IL-12Rb2–KO marrow, or mixed marrow consisting of WT CD3^+^ marrow cells and IL-12Rb2–KO non-CD3^+^ marrow cells (*n* = 5–6/group). Three weeks after transplantation, all mice were treated with 3 low-dose NHS–rmIL-12 treatments and followed for tumor growth. Significance determined by 2-way ANOVA. (**B**) Day 10 MOC22 tumors (*n* = 6) were dissociated, and the MFI of IL-12Rb2 expression was quantified on individual cell types via flow cytometry. (**C**) Heatmaps show average normalized expression of IL-12R subunits in different cell subsets identified in single-cell RNA-Seq data of 18 human papillomavirus–negative head and neck SCCs and 2 control-treated MOC22. Of note, only a few cells were classified as epithelial or keratinocyte in human single-cell RNA-Seq data (about 10 cells), as these experiments were performed using sorted immune cells.

Considering the widespread changes in T lymphocyte and myeloid populations observed during NHS–rmIL-12 treatment, we explored whether direct activation of T lymphocytes was sufficient for a complete treatment response or if concurrent modulation of the myeloid was additionally required. BM chimera studies were conducted to manipulate IL-12Rb2 expression within different immune cell compartments (Supplemental Figure 9, A and B). As expected, NHS–rmIL-12 treatment of irradiated mice engrafted with WT marrow resulted in significant tumor growth delay or cure, but no treatment effect was observed following treatment of irradiated mice engrafted with IL-12Rb2–KO BM (Figure 7A). However, growth delay but not cure of tumors was observed following treatment of mice engrafted with BM composed of WT CD3^+^ cells and IL-12Rb2–KO non-CD3^+^ cells. These results suggest that NHS–rmIL-12 directly activates T lymphocytes through binding of IL-12R to induce significant tumor growth delay, but they suggest that maximum treatment effect and cure of tumors requires IL-12Rb2 expression in the myeloid compartment. Although this chimera approach eliminated IL-12Rb expression on CD3^–^ lymphocytes as well as myeloid cells, B lymphocytes are unlikely to play a role in controlling MOC22 tumors following NHS–IL-12 treatment, given their paucity within the TME before or after treatment (Figure 3A and Supplemental Figure 10).

We next determined levels of IL-12Rb2 expression on different cell types within MOC22 tumors. The highest IL-12Rb2 expression was observed on macrophages, DCs, and Tregs, and to a lesser extent on FoxP3^–^ CD4 T lymphocytes (Figure 7B). These results support that therapeutic IL-12 can potentially modulate both the lymphoid and myeloid compartments within tumors. To determine if similar patterns of IL-12R expression are present within human head and neck SCCs, expression of IL-12Rb1 and IL-12Rb2 was analyzed in publicly available single-cell transcriptome data from 18 human papillomavirus–negative head and neck SCC (ref. 19 and Figure 7C). Similar to the expression patterns observed in mice, IL-12R is expressed on both lymphoid and myeloid cell subsets in human SCC. These results support that therapeutic IL-12 may enhance antitumor immunity in human SCCs through mechanisms similar to those we describe in murine SCCs.

## Discussion

In the present study, we performed a comprehensive characterization of molecular and cellular changes underlying IL-12–mediated tumor regression. We demonstrate the benefits of i.t. NHS–rmIL-12 relative to systemic administration. Our single-cell transcriptomic analysis demonstrates that an ongoing NHS–rmIL-12 treatment response involves reinvigoration of exhausted CD8^+^ T lymphocytes to gain effector qualities, polarization of the CD4 compartment toward a Th1 phenotype, and reduction of the number and suppressive capacity of Tregs through reduced expression of CTLA-4, IL-10, and CD39. Similarly, the myeloid compartment displayed a transition of M2-like macrophages toward an M1 phenotype and a reduction of *Tgfb1*-expressing monocytic-MDSC–like cells. Using chimera experiments, we show that maximum treatment efficacy of therapeutic IL-12 requires engagement of lymphoid and myeloid cells and that direct IL-12 activation of CD8^+^ T lymphocytes and Th1 polarization of CD4^+^ T lymphocytes in the context of an unaffected myeloid compartment is insufficient to control tumors. This potentially novel finding demonstrates that modulation of both lymphoid and myeloid cells is required for tumor cure. Furthermore, similar IL-12R expression patterns between human and mouse immune cells indicate that IL-12 may have similar effects on immune cell subsets across organisms.

By delivering dose-reduced NHS–rmIL-12 with an i.t. technique, greater i.t. IFN-γ levels were achieved, along with reduced systemic IFN-γ levels compared with peripheral s.c. treatment. These data support that i.t. administration of dose-reduced NHS–rmIL-12 may reduce the risk of elevated peak IFN-γ levels in circulation after treatment and, thus, reduce the risk of treatment-related adverse events. Our data also support the work of others demonstrating positive antitumor effects of i.t. IL-12 delivery approaches in preclinical models of cancer (12, 20). In our model, i.t. administration of NHS–rmIL-12 appears effective despite tumors lacking significant regions of necrosis at the time of treatment initiation. I.t. NHS–rmIL-12 demonstrated superior induction of antitumor immunity and tumor control when compared with free i.t. rmIL-12, suggesting that the NHS further retains this cytokine within the TME.

Myeloid populations, including DCs, play a critical role in type I IFN responses and induction of antitumor immunity (21). Endogenous production of IL-12 by DCs in response to increased IFN-γ levels contribute to enhancement of antitumor immunity after immune checkpoint blockade (21). It is possible that similar IFN-γ–dependent positive feedback mechanisms occur following treatment with exogenous IL-12 cytokine, and this could be explored with antibody or genetic depletion of IFN-γ signaling or CD8^+^ cells during NHS–rmIL-12 treatment. Such studies further clarifying the requirement for cross talk between myeloid and lymphoid population in effective responses to NHS–rmIL-12 are warranted.

Limitations of our study exist. Our BM chimera studies do not allow us to determine which myeloid subsets are specifically responsible for the observed enhancement of CD8-dependent effector immunity. Similarly, a contribution of CD3^–^ lymphocytes was not ruled out in the chimera experiments, although the presence of very low numbers of B lymphocytes within the TME makes this highly unlikely. Mechanism of treatment effect was studied in tumors that lacked significant regions of tumor necrosis at the time of treatment. It is possible that different results could be observed following treatment of larger tumors where NHS–rmIL-12 is focally concentrated in regions of necrosis present at the start of treatment. We demonstrated that NHS–rmIL-12 reduces macrophage-suppressive capacity and polarizes subsets toward an M1 phenotype, but this does not rule out that modulation of other myeloid cells, including DCs, plays an important role. Although we demonstrated robust tumor control and cure in the immunogenic MOC22 model that was used for mechanistic studies, less robust tumor growth control was observed with i.t. NHS–rmIL-12 treatment in the less antigenic MOC1 and MC38 models, though greater tumor control was observed compared with systemic s.c. treatment, suggesting that this finding is generalizable. We did not explore whether tumor control could be enhanced with NHS–rmIL-12 in combination with other immunotherapies such as immune checkpoint blockade in these more resistant models. Additive or synergistic effects of NHS–rmIL-12 combined with immune checkpoint blockade or histone deacetylase inhibition in numerous syngeneic models of cancer have been demonstrated by others (22–24). These results suggest that single-agent i.t. NHS–IL-12 may be more effective in the premalignant or early-stage cancer setting, whereas combination immunotherapy including i.t. NHS–IL-12 should be considered for more advanced tumors. Further clinical investigation of i.t. NHS–IL-12 is warranted.

## Methods

### Cell lines, animal studies, and reagents.

Original stocks of genomically characterized (25) MOC22 and MOC1 cells were gifts from Ravindra Uppaluri (Dana-Farber Cancer Institute, Boston, Massachusetts, USA) and maintained in culture as described (26). MC38 cell lines were obtained commercially from Kerafast. Cells were used for all experiments at low passage number (<30 passages), maintained in sterile culture conditions and serially tested for mycoplasma (Lonza MycoAlert Mycoplasma Detection Kit). Tumors were established by s.c. injection of tumor cells (1 × 10^6^ to 5 × 10^6^) in Matrigel (30% by volume). WT C57BL/6 (B6) mice aged 6–8 weeks were purchased from Taconic. IL-12Rb2–KO B6;129S1-*Il12rb2^tm1Jm^*/J mice and CD45.1 transgenic (B6.SJL-*Ptprc^a^ Pepc^b^*/BoyJ) mice were purchased from The Jackson Laboratory. Tumor volume was calculated as: (length^2^ × width)/2. NHS–rmIL-12 was obtained from EMD Serono through a Cooperative Research and Development Agreement with the National Cancer Institute. NHS–rmIL-12 was diluted in sterile 1× PBS for use via s.c. or i.t. injection. In vivo grade rmIL-12 was purchased from Peprotech. Low endotoxin in vivo antibodies specific for PD-1 (clone RPM1-14), CD8 (clone YTS 169.4), CD4 (clone GK1.5), NK1.1 (clone PK136), and CD25 (clone PC61) and isotype control antibody clones 2A3 and HRPN were purchased from BioXCell. For cellular depletion studies, 200 μg of antibody was administered i.p. twice weekly for 3 weeks.

### ELISA.

Human IgG ELISA and murine IFN-γ ELISA kits were purchased from R&D Systems and used per manufacturer recommendations. To prepare tumor supernatants for ELISA where indicated, tumors were homogenized in 1× PBS using the gentleMACS Dissociator (Miltenyi Biotec) per manufacturer recommendations.

### Flow cytometry.

Tumor tissues were processed into single-cell suspensions by mincing, as well as chemical (Murine Tumor Dissociation Kit, Miltenyi Biotec) and mechanical (gentleMACS Dissociator) dissociation, per manufacturer recommendations. Suspensions were filtered through a 100 μm filter and washed with 1% BSA in PBS prior to blocking nonspecific staining with anti-CD16/32 (BioLegend) antibody. Cell surface staining was performed using fluorophore-conjugated anti–mouse CD45.2 (clone 104), CD3 (clone 145-2C11), CD4 (clone GK1.5), CD8 (clone 53-6.7), CD31 (clone 390), PDGFR (clone APA5), PD-L1 (clone 10F.9G2), H2-K^b^ (clone AF6-88.5), CD107a (clone 1D4B), PD-1 (clone RMP1-30), CD11b (clone M1/70), Ly6G (clone 1A8), Ly6C (clone HK1.4), CTLA-4 (clone 9H10), CD39 (clone Duha59), CD11c (clone N418), F4/80 (clone BM8), and CD206 (clone C068C2) from BioLegend, and IL-12Rb2 (clone 305719) from R&D Systems. FoxP3^+^ Treg staining performed with the mouse Treg Staining Kit #1 (eBioscience) as per manufacturer’s protocol. Cell viability was assessed via staining with Sytox (Thermo Fisher Scientific) or Zombie (BioLegend) dyes. All analyses were performed on a BD Fortessa analyzer running FACSDiva software and interpreted using FlowJo V.X10.0.7r2.

### Impedance analysis.

Real-time impedance analysis was performed using the xCELLigence Real-Time Cell Analysis platform per manufacturer recommendations and as previously described (27). Triton X-100 (0.2%) was used as a positive control for complete cell lysis. Percent loss of cell index was calculated as: 1 − (experimental cell index/control cell index) for a given time point. 

### TIL culture.

Tumors were harvested, minced into 1 mm fragments, and placed into a 24-well place with RPMI1640-based media supplemented with 10% FBS and rmIL-2 (100U/mL). Every 2–3 days, half of the media volume was replaced. Tumor fragments were removed on day 4, and TIL were harvested for experimental use on day 12. Negative magnetic isolation with the Stemcell EasySep Mouse T cell isolation Kit, used per manufacturer recommendations, was used to deplete non-T lymphocytes before experimental use.

### T lymphocyte functional assays.

Cultured TIL from tumors were cocultured with irradiated (18 Gy) WT B6 splenocytes pulsed with 1 μg/mL of peptide at a 2:1 APC/TIL ratio for 24 hours. Negative controls included coculture of TIL with APC pulsed with no or irrelevant (OVA) peptide. TIL were exposed to 1× PMA/Iono (Thermo Fisher Scientific) as a positive control. Murine IFN-γ ELISpot kits were purchased from R&D Systems and used per manufacturer recommendations. Spot counts were measured on an Immunospot ELISpot plate reader from Cellular Technology.

### T lymphocyte suppression assays.

T cells isolated from WT B6 mouse spleens via negative magnetic selection (EasySep Mouse T cell Isolation Kit, Stemcell Technologies) were stained with 5 μmol/L carboxyfluorescein succinimidyl ester (CFSE; MilliporeSigma) and stimulated using plate-bound CD3 (clone 145-2C11, eBioscience) and CD28 (clone 37.51, eBioscience) antibodies as described (28). To isolate tumor immune cells, digested tumor single-cell suspensions were first enriched for lymphocytes using a 40/80% isotonic Percoll (MilliporeSigma) gradient (centrifuged at 325*g* for 23 minutes at room temperature), followed by magnetic isolation for Tregs (EasySep Mouse CD4^+^CD25^+^ Regulatory T cell Isolation Kit II), macrophages (EasySep Mouse F4/80^+^ Selection Kit), or neutrophilic cells (EasySep Mouse Neutrophil Enrichment Kit) using reagents from Stemcell per manufacturer recommendations. T cells and isolated tumor infiltrating cells were then cocultured at a 1:2 ratio for 72 hours. Flow cytometry was used to quantify CFSE dilution. Proliferation was quantified as the average number of divisions for all cells in the culture (division index) using FlowJo software. IFN-γ concentration in culture supernatant was determined by ELISA. Media for all functional immune assays consisted of RPMI1640 supplemented with 10% FCS, 2 μmol/L β-ME, HEPES, nonessential amino acids, glutamine, and antibiotics.

### BM chimera studies.

To ablate endogenous BM, WT B6 mice were irradiated to 9 Gy using an X-RAD 320 (Precision X-Ray). After a 6-hour rest period, mice were transplanted with 4 × 10^6^ donor BM cells via i.v. tail vein injection. BM cells were flushed from the femurs of donor WT B6 or IL-12Rb2–KO mice. Marrow was subjected to RBC lysis (Thermo Fisher Scientific) and filtered (30 μM). For BM mixing studies, untouched WT BM CD3^+^ cells were isolated from WT B6 donor marrow with the EasySep Mouse T cell Isolation Kit (Stemcell) from Stemcell used per manufacturer recommendations. Untouched IL-12Rb2–KO CD3^–^ cells were isolated from IL-12Rb2–KO donor marrow by using positive selection to remove CD3^+^ cells with a PE-conjugated CD3 primary antibody (BioLegend), followed by positive selection of PE-labeled cells with the EasySep Mouse PE Positive Selection Kit II (Stemcell Technologies) used per manufacturer recommendations. Transplanted mixed BM consisted of 10% CD3^+^ and 90% CD3^–^ cells. Mice were allowed 3 weeks to engraft new marrow before being transplanted with MOC22 cells and used to in vivo studies.

### Single-cell capture and sequencing.

Whole tumors were digested into single-cell suspensions, washed in 1× PBS, filtered (70 μm) and quantified using acridine orange/propidium iodide (AO/PI) staining on a Cellometer Auto 2000 (Nexcelom). Cells were concentrated to 1000 cells/μL and loaded onto the Chromium Controller (10X Genomics) with a target of 10,000 cells per sample. Cells were mixed with barcoded gelbeads and 3′ GEM Kit v3 reagents, and single-cell capture was performed. Following reverse transcription, cDNA was amplified, and sequencing libraries were constructed according to the manufacturer’s recommendations. Each DNA library was loaded into a sequencing lane on a NovaSeq system (Illumina) and was sequenced with pair-end reads of 75 bp. Demultiplexing was done allowing 1 mismatch in the barcodes. UMI counts were obtained using Cell Ranger (v6.0.2; ref. 29) with default parameters.

### Single-cell transcriptome analysis.

Single-cell transcriptome analysis was performed using R (R Core Team, 2020) and the R package Seurat (30) using filtered UMI count matrices. The following criteria were applied to filter cells — i.e., cells satisfying 1 or more of the following conditions were removed: cells with (a) ≥ 25% Hba or Hbb UMI counts, (b) ≥ 25% mitochondrial UMI counts, (c) 250 > genes with nonzero UMI counts > 5000, or (d) sum of UMI over all genes < 500. In addition, genes detected in fewer than 10 cells were removed. If not stated otherwise, Seurat functions were used with default parameters. UMI counts were normalized (Seurat: NormalizeData), variable feature were identified (Seurat: FindVariableFeatures), the data was scaled (Seurat: ScaleData[vars.to.regress = c(‘nCount_RNA’)]), and a PCA was performed using the variable features (Seurat:RunPCA). Harmony (31) was applied to (Seurat: RunHarmony) to integrate individual data sets. Of note, Harmony integration was omitted at during clustering of cell types to better preserve treatment effects. UMAP embeddings were generated (Seurat: RunUMAP) using “harmony” reduction when available and “pca” otherwise. Similarly, graph-based clustering was performed using “harmony” reduction when available and “pca” otherwise (Seurat: FindNeighbors and Seurat: FindClusters). Differential gene expression was performed using default parameters (Seurat: FindMarkers[logfc.threshold = 0.25, min.pct = 0.1]). Cell type identification in human single-cell RNA-Seq data was performed using R packages SingleR and celldex (32) using the Blueprint reference data.

### Reactome pathway enrichment analysis.

Reactome pathway (33) enrichment analysis was performed using the R package clusterProfiler (34) using genes upregulated in NHS–rmIL-12–treated cells based on fold change. Upregulated genes were provided to the function clusterProfiler:enrichPathway using organism = “mouse”. The resulting enrichment terms were visualized using enrichplot:dotplot (https://yulab-smu.top/biomedical-knowledge-mining-book/).

### Statistics.

All murine genomic data are available through the GEO repository (GSE186636). Reanalyzed human single-cell transcriptomic data are available through the original publication (19). Differences between means were obtained either with an unpaired, 2-tailed Student *t* test for individual comparisons and 1-way or 2-way ANOVA with Holm–Sidak correction for multiple-group comparisons. The Wilcoxon signed-rank test was used to compared nonparametric sets of data. Quantification of necrotic surface area of H&E-stained slides was performed using the annotation function of QuPath V0.2.2. Data are shown as mean ± SD. A *P* value less than 0.05 was considered significant. Analyses were performed using GraphPad Prism V.7.

### Study approval.

All animal studies were conducted after full review and approval by the NIH Animal Care and Use Committee (protocol no. 1364).

## Author contributions

YH, WKM, JLG, JS, SRG, and CTA conceived and designed the studies. YH, YR, XY, AS, JBM, and CTA generated data, key reagents, and samples. YH, YR, WKM, AS, JBM, JLG, JS, CS, and CTA analyzed and interpreted the data. YH, YR, XY, WKM, AS, JBM, JLG, JS, SRG, CS, and CTA wrote and revised the manuscript. CS and CTA are co–senior authors; order of authors was decided based on the fact that CTA conceived of and was responsible for oversight of all experimental work. All authors approved the final version of the manuscript.

## Supplementary Material

Supplemental data

## Version 1 03/08/2022

Electronic publication
