# Supplementary material for: Cure of syngeneic carcinomas with targeted IL-12 through obligate reprogramming of lymphoid and myeloid immunity
Source: JCI Insight. 2022 Mar 8;7(5):e157448. doi: 10.1172/jci.insight.157448 (PMC8983130; doi:10.1172/jci.insight.157448)
Supplement: Supplemental data [file jciinsight-7-157448-s020.pdf]

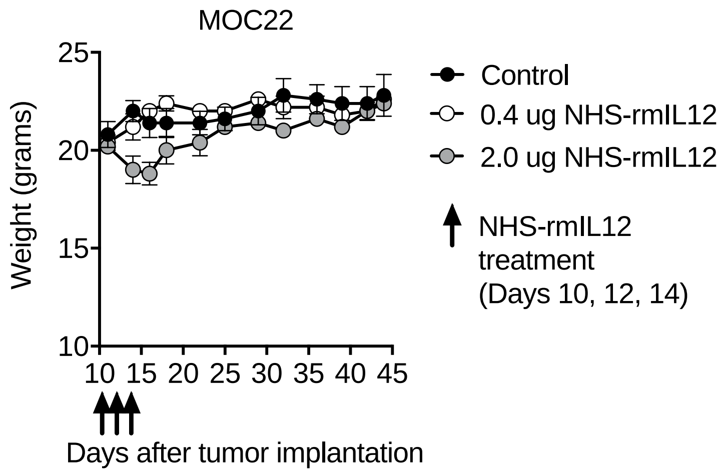

Supplementary Figure 1– Mouse weights with NHS-rmIL12 treatment

Mice bearing established MOC22 tumors were treated with high-dose (2.0 ug) or low-dose (0.4 ug) peripheral subcutaneous NHS-rmIL12. Mice were weighed at least twice weekly.

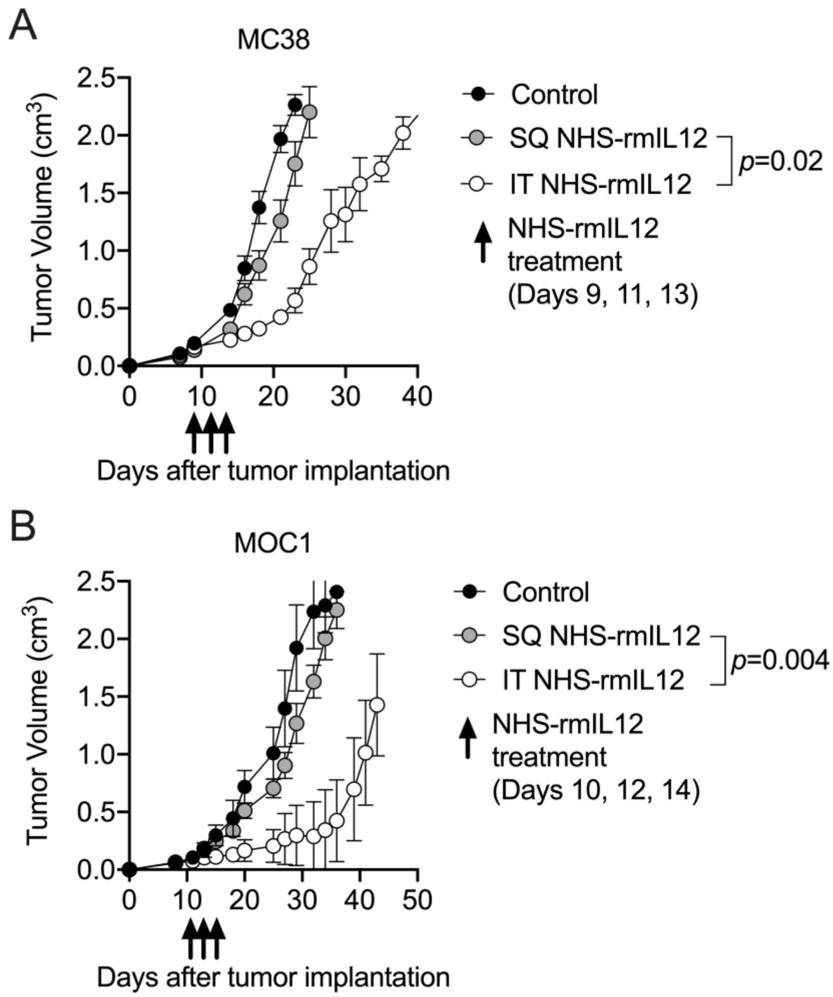

Supplementary Figure 2 – Dose-reduced tumor targeted NHS-rmIL12 induces growth delay in multiple syngeneic models of carcinoma

Mice bearing established (A) MC38 colon or (B) MOC1 oral carcinomas (*n*=10/group) were treated with peripheral subcutaneous or intratumoral low-dose (0.4 ug) NHS-rmIL12. P-values indicating the significance of differences between summary growth curves were determined using a two-tailed paired t-test.

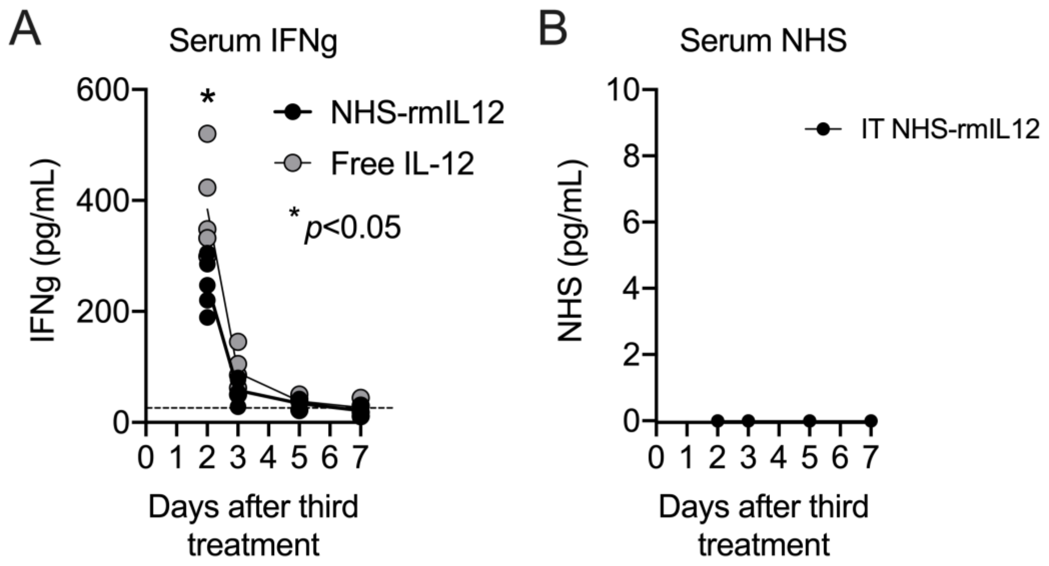

Supplementary Figure 3 – NHS-rmIL12 treatment results in reduced systemic interferon levels compared to free IL-12

A, a time course of IFNg concentration was measured in the serum of MOC22 tumor-bearing mice (*n*=5/group) via ELISA following three low-dose (0.4 ug) intratumoral NHS-rmIL12 or molar-equivalent (0.29 μg) free IL-12 treatments. *indicates a significant difference (p<0.05) between NHS-rmIL12 and free IL-12 determined by a two-tailed Student t test.

B, a time course of NHS antibody concentration was measured in the serum of MOC22 tumor-bearing mice (*n*=5/group) via ELISA following three low-dose (0.4 ug) intratumoral NHS-rmIL12 treatments.

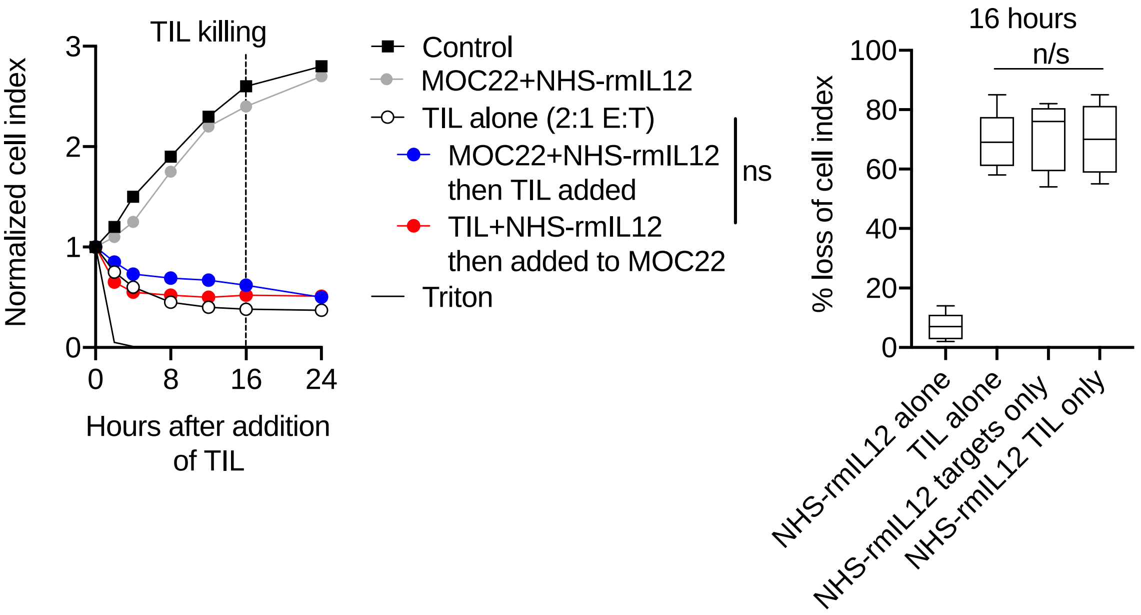

Supplementary Figure 4 – NHS-rmIL12 does not directly alter tumor cell viability *in vitro*

Proliferation and viability of MOC22 cells in culture alone (black line) or upon exposure to NHS-rmIL12 (10 ng/mL*; gray line) was measured via real time impedance analysis. The ability of tumor infiltrating lymphocytes from MOC22 tumors to kill MOC22 cells at baseline (white line) or following exposure of the MOC22 cells (blue line) or TIL (red line) to NHS-rmIL12 was also determined. A representative impedance plot is shown on the left, and the cumulative results of loss of impedance at 16 hours after the addition of effectors in three independent experiments are shown on the right.

*Based upon conversion of 1 mg tissue = 1 μL volume, with a maximum drug concentration of 10 pg/mg tissue in tumor after treatment with low-dose (0.4 ug) NHS-rmIL12 based upon results from experiments detailed in **Figure 1F**.

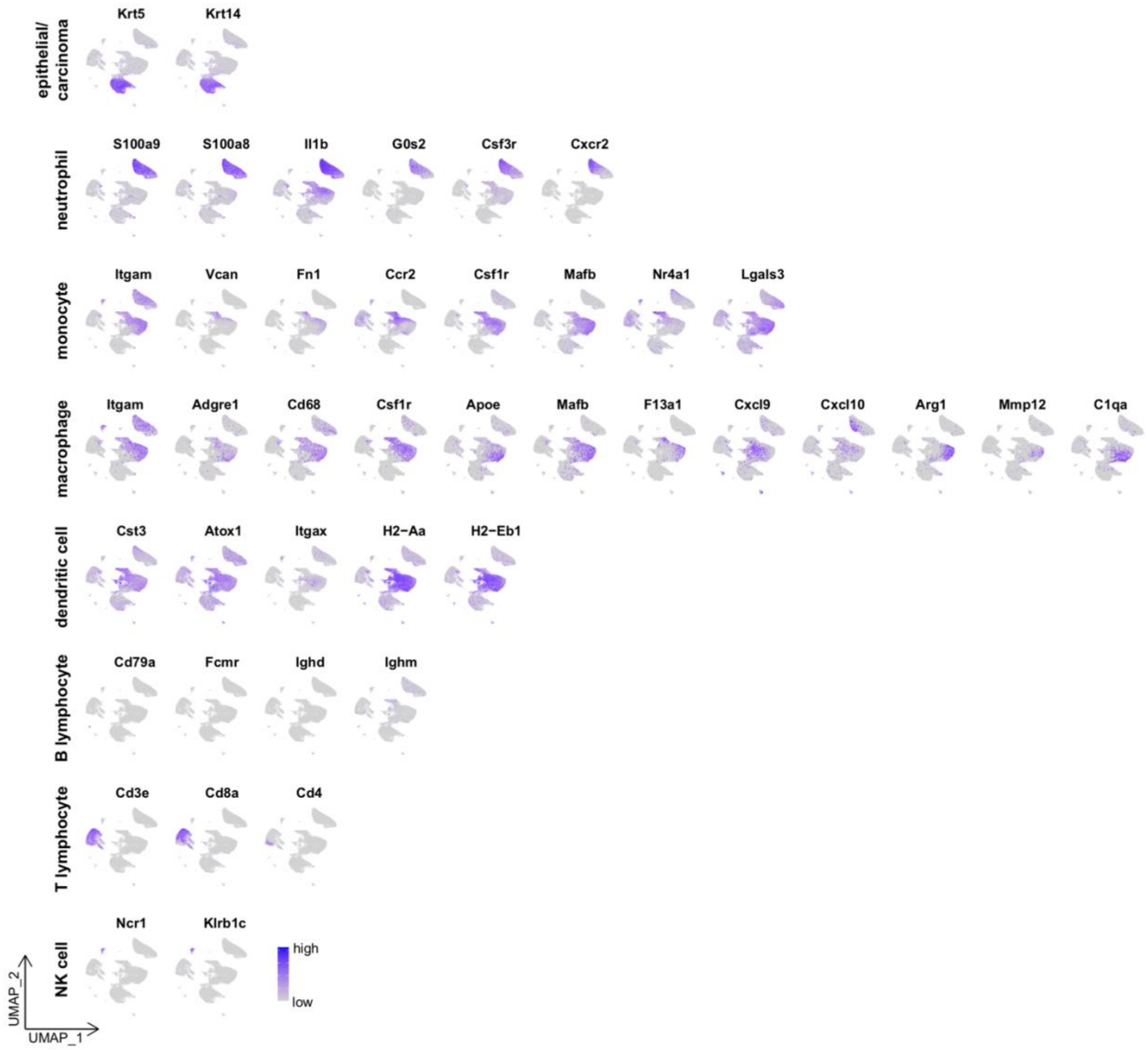

Supplementary Figure 5 – Featureplot demonstrating cell type-specific marker gene expression

Featureplot is shown of UMAP embedded cell lineage-specific gene expression.

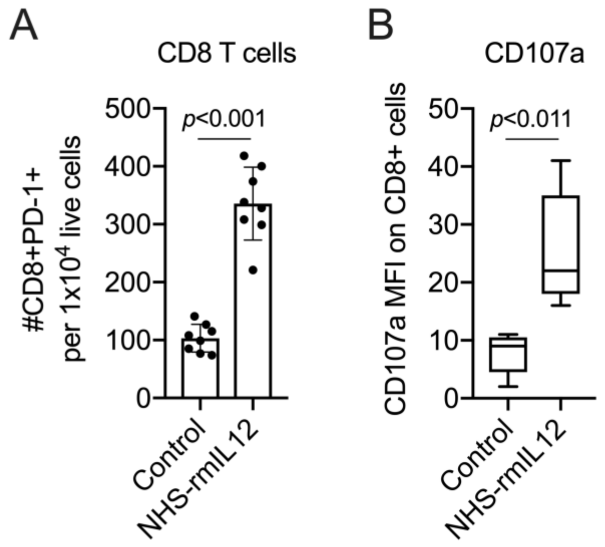

Supplementary Figure 6 – Increased tumor PD-1 positive CD8+ T lymphocytes validated by flow cytometry

A, quantification of PD-1 positive CD8+ T-lymphocytes from tumors treated with NHS-rmIL12 or PBS control (*n*=8/group) measured by flow cytometry.

B, MFI of cell surface CD107a on CD8+ T-lymphocytes (*n*=8/group) measured by flow cytometry. P-value determined by two-tailed Student t test.

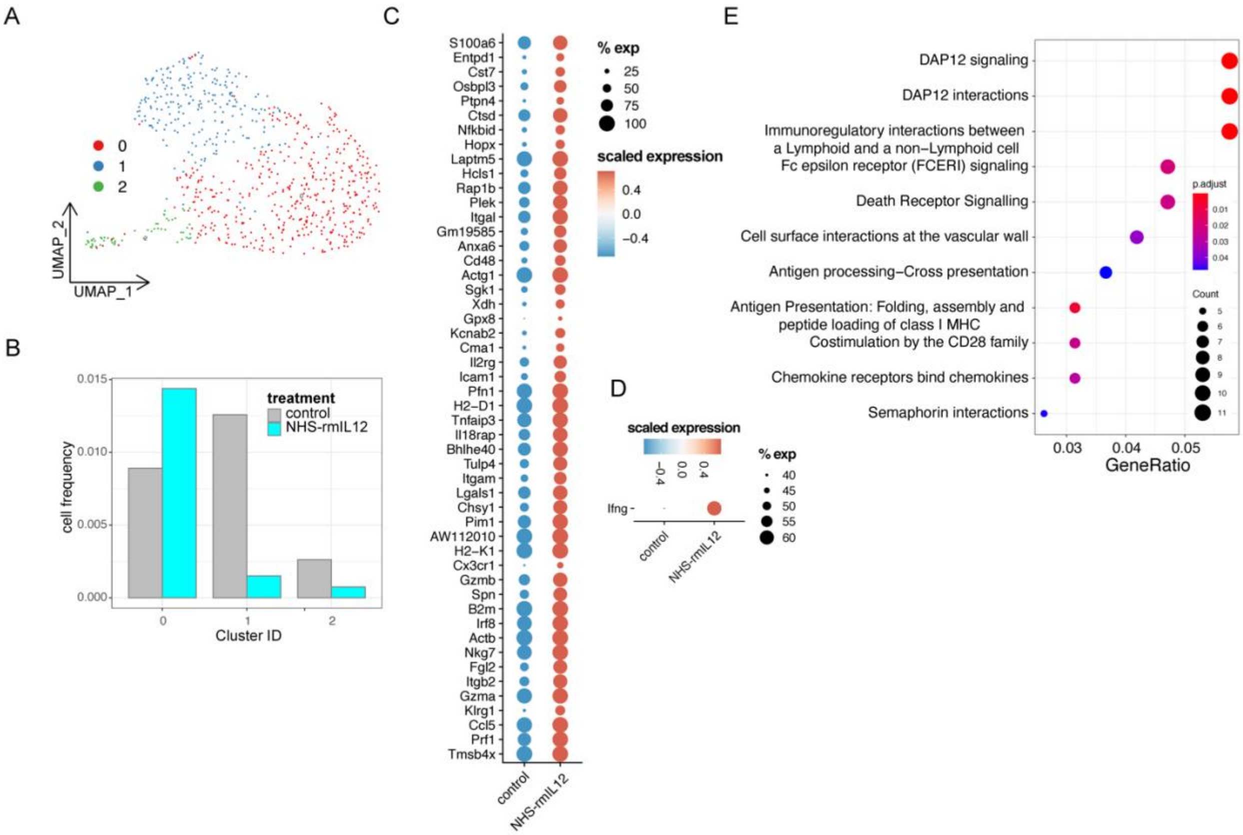

Supplementary Figure 7 – NK cells are activated after NHS-rmIL12 treatment

A, UMAP embedding of NK cells colored by cluster identity.

B, barplots demonstrating the frequency of each NK subcluster relative to all cells from tumors treated with NHS-rmIL12 or PBS control.

C, dot plot showing expression of the top differentially expressed genes across all NK cells from tumors treated with PBS control or NHS-rmIL12. Circle color corresponds to scaled average expression; circle size denotes fraction of cells with non-zero gene expression of corresponding gene.

D, dot plot of NK cell IFNg average gene expression in tumors treated with PBS control or NHS-rmIL12.

E, dot plot showing Reactome terms enriched in genes upregulated by NHS-rmIL12-treatment within all NK clusters

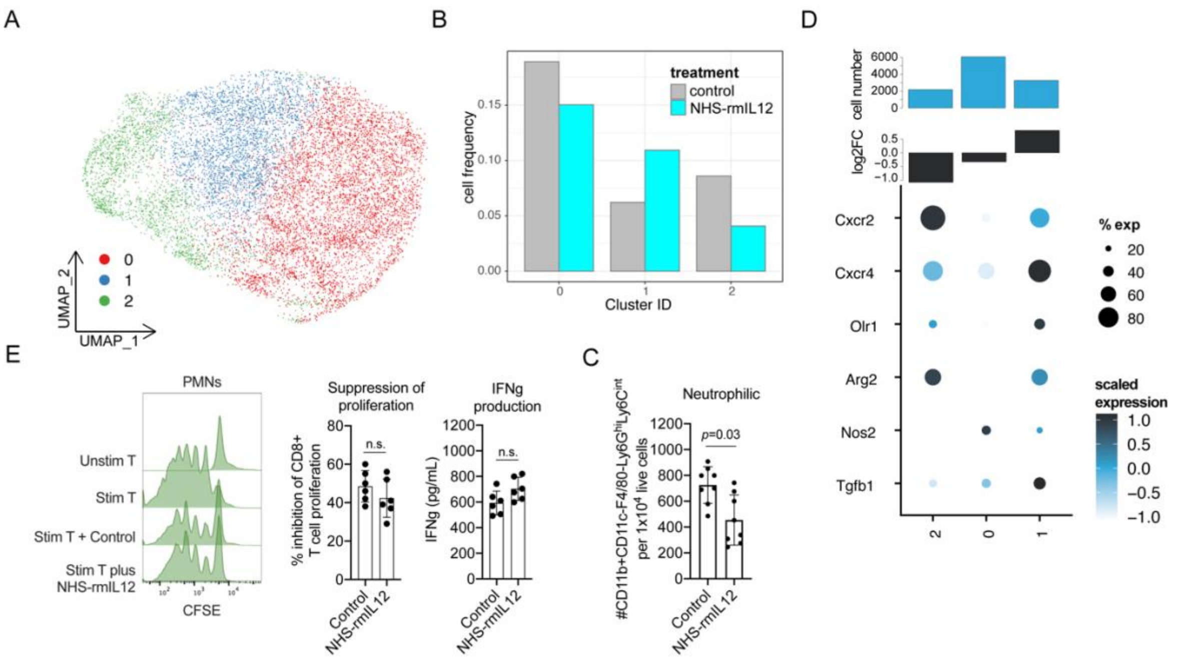

Supplementary Figure 8 – Neutrophilic cells are relatively unaltered by NHS-rmIL12 treatment

A, UMAP embedding of neutrophilic cells colored by cluster identity.

B, barplots demonstrating the frequency of each neutrophil subcluster relative to all cells from tumors treated with NHS-rmIL12 or PBS control.

C, quantification of CD11b and Ly6G positive neutrophils from tumors treated with NHS-rmIL12 or PBS control measured by flow cytometry. P-value determined by student's t-test.

D, dot plot showing expression of select neutrophil-related genes across neutrophil clusters sorted by fold change in relative cell abundance comparing cells from NHS-rmIL12- to control-treated tumors (lower bar graph). Circle color corresponds to scaled average expression; circle size denotes fraction of cells with non-zero gene expression of corresponding gene. Top bar graph represents total cell number.

E, Ly6G positive cells were isolated from tumors treated with NHS-rmIL12 or PBS control via magnetic selection and co-cultured with CFSE-labelled WT T lymphocytes stimulated with

CD3/28 antibodies. Representative CFSE histograms are shown, T lymphocyte proliferation was measured by flow cytometry and IFNg production was measured by ELISA. P-value determined by two-tailed Student t test.

n.s., not significant

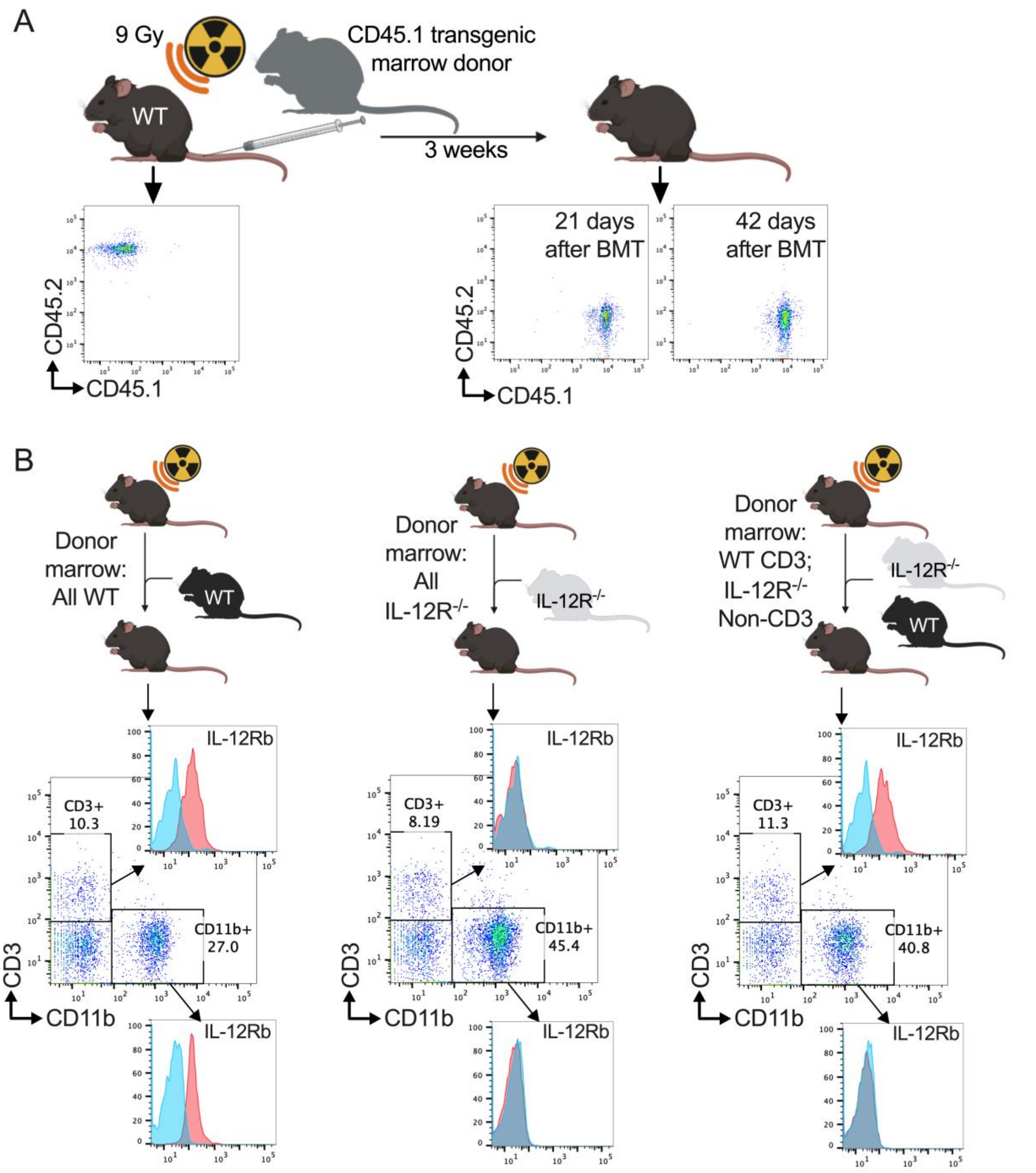

Supplementary Figure 9 – Validation of bone marrow chimera methods

A, WT B6 mice were irradiated to 9Gy and transplanted with bone marrow from CD45.1 transgenic B6 mice. After 3 or 6 weeks, flow cytometry was used to assess for the presence of endogenous CD45.2 immune cells or transplanted CD45.1 immune cells in the peripheral blood.

B, WT B6 mice were irradiated to 9Gy and transplanted with bone marrow from WT B6 mice, IL-12Rb2 knockout mice, or a mixture of WT CD3+ marrow cells and IL-12Rb2 knockout non-CD3+ marrow cells. After three weeks, flow cytometry was used to assess for the presence of IL-12Rb2 expression on CD3+ or CD11b+ cells in the peripheral blood.

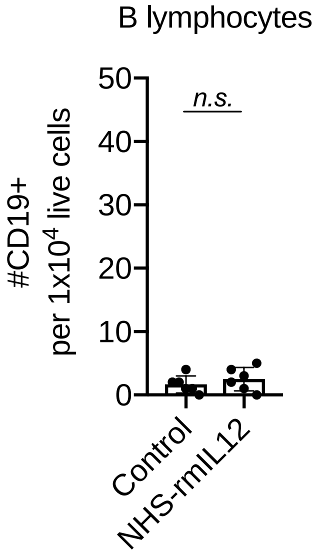

Supplementary Figure 10 – MOC22 tumors harbor few CD19+ B lymphocytes

Quantification of CD19 positive cells from tumors treated with NHS-rmIL12 or PBS control (*n*=6/group) measured by flow cytometry.

Supplementary Table I. Gene markers for cell-type modules.

cell_type	gene_id
macrophages	Itgam
macrophages	Adgre1
macrophages	Cd68
macrophages	Siglec1
macrophages	Csf1r
macrophages	Apoe
macrophages	Mafb
macrophages	Cx3xr1
macrophages	F13a1
macrophages	Lgals3
macrophages	Cxcl9
macrophages	Cxcl10
macrophages	Arg1
macrophages	Mmp13
macrophages	Mmp12
M1_macrophages	Cd38
M1_macrophages	Nos2
M1_macrophages	Cd40
M1_macrophages	Cd86
M1_macrophages	Marco
M1_macrophages	Socs3
M1_macrophages	Il12b
M1_macrophages	Ptgs2
M1_macrophages	Il23a
M1_macrophages	Ido1
M2_macrophages	Mrc1
M2_macrophages	Cx3cr1
M2_macrophages	Cd163
M2_macrophages	Retnla
M2_macrophages	Socs2
M2_macrophages	Irf4
M2_macrophages	Cxcl13
M2_macrophages	Ccl12
M2_macrophages	Ccl24
M2_macrophages	Klf4
monocyte	Itgam
monocyte	Ly6c1
monocyte	Vcan
monocyte	Fn1
monocyte	Ccr2
monocyte	Csf1r
monocyte	Mafb

monocyte	Nr4a1
monocyte	Lgals3
dendritic_cell	Cst3
dendritic_cell	Atox1
dendritic_cell	Nccrp1
dendritic_cell	Itgax
